# Are sedentary behavior and physical activity independently associated with cardiometabolic benefits? The Hispanic Community Health Study/Study of Latinos

**DOI:** 10.1186/s12889-020-09497-5

**Published:** 2020-09-14

**Authors:** Yasmin Mossavar-Rahmani, Simin Hua, Qibin Qi, Garrett Strizich, Daniela Sotres-Alvarez, Gregory A. Talavera, Kelly R. Evenson, Marc D. Gellman, Mark Stoutenberg, Sheila F. Castañeda, Linda C. Gallo, Krista M. Perreira, Lisa A. P. Sanchez-Johnsen, Robert C. Kaplan

**Affiliations:** 1 Department of Epidemiology & Population Health, Albert Einstein College of Medicine, 1300 Morris Park Avenue, Belfer Bldg, 1312C, Bronx, New York, 10461 USA; 2 Collaborative Studies Coordinating Center, Department of Biostatistics, Gillings School of Global Public Health, University of North Carolina, Chapel Hill, North Carolina 27516 USA; 3 Department of Psychology, San Diego State University, San Diego, California 91910 USA; 4 Department of Epidemiology, Gillings School of Global Public Health, University of North Carolina, Chapel Hill, North Carolina 27599 USA; 5 Department of Psychology, University of Miami, Coral Gables, Florida 33136 USA; 6 Department of Health & Human Performance, University of Tennessee, Chattanooga, TN 37403 USA; 7 Department of Social Medicine, University of North Carolina School of Medicine, Chapel Hill, North Carolina 27599 USA; 8 Department of Family Medicine, Rush University Medical Center, Chicago, IL 60612 USA; 9 Fred Hutchinson Cancer Research Center, Division of Public Health Sciences, Seattle, Washington 98109 USA

**Keywords:** Cohort study, Moderate-to-vigorous physical activity, Sedentary behavior, Cardiometabolic biomarkers, Hispanics/Latinos

## Abstract

**Background:**

Whether physical activity can reduce cardiometabolic risk particularly in understudied populations such as US Hispanics/Latinos is of public health interest. We prospectively examined the association of physical activity and cardiometabolic biomarkers in *n* = 8049 participants of the Hispanic Community Health Study/Study of Latinos, a community-based cohort study of 16,415 adults aged 18–74 yr who self-identified as Hispanic/Latino from four US urban centers.

**Methods:**

We assessed physical activity using accelerometry in 2008–2011 at visit 1. We assessed cardiometabolic biomarkers twice: once at visit 1 and collected a second measure in 2014–2017 at visit 2. We used survey linear regression models with changes in cardiometabolic markers as the dependent variables and quartiles of sedentary behavior or whether adults met guidelines for moderate-to-vigorous physical activity as the independent variables.

**Results:**

In normoglycemic adults without cardiovascular disease, but not in adults with evidence of cardiometabolic disease, those who were in the lowest quartile for sedentary behavior (< 10.08 h/day) had a significant decline in mean LDL-cholesterol of − 3.94 mg/dL (95% CI: − 6.37, − 1.52) compared to adults in the highest quartile (≥13.0 h/day) who exhibited a significant increase in LDL-cholesterol of 0.14 mg/dL (95% CI, − 2.15,2.42) over the six year period (*P* < 0.02 in fully adjusted models.) There was also a trend toward lower mean increase in HbA1c comparing the lowest with the highest quartile of sedentary behavior. Overall regardless of glycemic level or evidence of cardiometabolic disease, adults who met guidelines for moderate-to-vigorous physical activity at visit 1, had significantly lower mean increases in level of fasting glucose compared to adults not meeting guidelines in fully adjusted models.

**Conclusions:**

In this cohort of Hispanics/Latinos, being free of cardiometabolic disease and having low levels of sedentary behavior were associated with health benefits. Among all adults regardless of cardiometabolic disease, meeting guidelines for moderate-to-vigorous physical activity was associated with health benefits. Overall these data suggest that an active lifestyle may blunt the association of advancing age with worsening cardiometabolic risk factors.

## Background

In the last few decades, physical activity levels of Americans have decreased even though physical activity is associated with maintaining good health over the lifecycle [1, 2]. Major benefits of physical activity include protection against heart disease and diabetes. However knowledge as to what aspects of cardiometabolic parameters are affected is limited, especially among Hispanics/Latinos who are the largest minority population in the US [3]. Several aspects of behavior and predisposition to disease may differ in Hispanics/Latinos versus others, for example a high burden of obesity and diabetes [4]. Hispanics/Latinos also have a relatively high level of work- and transportation-related physical activity, which may be qualitatively different from other forms of exercise such as leisure time and thus may have a different relationship with health outcomes [5, 6].

According to the 2018 US Guidelines for Americans, adults should engage in at least 150 min to 300 min a week of moderate-intensity, or 75 min to 150 min a week of vigorous-intensity aerobic physical activity, or an equivalent combination of moderate-to-vigorous activity (MVPA) [7]. Physical activity helps protect against heart disease and factors related to the progression of type 2 diabetes by helping to reduce the risk factors of high blood pressure, body weight, blood lipids (cholesterol), and elevated hemoglobin A1c in individuals with type 2 diabetes. The beneficial effects on blood glucose (indicated by hemoglobin A1c) may also reduce other complications of type 2 diabetes. Moderate-intensity activity for at least 150 min a week plus 2 days a week of muscle-strengthening activities help to substantially lower the risk of heart disease. Three hundred minutes or more of moderate-intensity activity a week may lead to even greater benefit.

Sedentary behavior which is broadly defined as energy expenditure (<= 1.5 METs) and a posture of prolonged sitting and reclining) [8] on the other hand, may raise cardiometabolic risk, due to effects of skeletal muscle contraction on metabolic function and the homeostatic regulation of body weight and fat mass which is based on loading, known as graviostat [9, 10]. According to the graviostat concept, the body’s intrinsic weight sensor senses less weight during sedentary behavior, and in turn the body’s regulatory mechanisms compensate by increasing body fat to keep body weight at a set point. Diabetes has a strong association with sedentary behavior [11–13]. Even light activity such as walking to interrupt sedentary behavior may be beneficial [14]. Only a few national guidelines set targets for sedentary behavior (e.g. Australia) [15]; however additional data describing the dose-response patterns linking sedentary behavior levels with adverse health outcomes, independent of MVPA could motivate more countries to address guidelines with respect to sedentary behavior levels. Reduction of sedentary behavior using intervention strategies such as preventing uninterrupted prolonged sitting by including breaks may be a particularly useful complementary strategy beyond promotion of MVPA. Public health strategies that will empower individuals to reduce sedentary behavior (e.g., increased walking, standing at work, taking breaks from sitting) are different than those that promote MVPA.

Our prior work indicated in the Hispanic Community Health Study/Study of Latinos (HCHS/SOL) that sedentary behavior was cross-sectionally associated with several cardiometabolic risk factors even among adults with high levels of MVPA and among adults meeting physical activity guidelines. In addition, adults who were older, women, and those with higher income had a higher prevalence of sedentary behavior [16, 17].

Here we extend this cross-sectional work by exploring prospectively the associations of sedentary behavior and MVPA with changes in cardiometabolic biomarkers six years later. We investigated whether there were changes in body mass index (BMI), waist circumference, systolic/diastolic blood pressure, low density lipoproteins (LDL) and high density lipoproteins (HDL) cholesterol, triglycerides, 2 h post-load glucose, homeostatic model assessment of insulin resistance (HOMA-IR), fasting plasma glucose, fasting insulin, hemoglobin A1c (HBA1c); whether these changes were associated with sedentary behavior and according to meeting or not meeting the physical activity guidelines for MVPA; how these changes differed by subgroups based on glycemic level; and whether there was evidence for variation in these findings across age (< 60 vs ≥60 years old), sex, and Hispanic/Latino groups. We investigated insulin and insulin resistance because they are risk factors for cardiovascular disease and mortality, [18] while other study outcomes are widely accepted targets for cardiovascular preventive treatments. Finally we explored sex differences in cardiometabolic benefits from physical activity [19]. We hypothesized that sedentary behavior and MVPA were independently associated with cardiometabolic biomarkers and that pre-existing cardiovascular disease or diabetes may attenuate the beneficial association of physical activity with cardiometabolic biomarkers, a question that has not been well-addressed in Hispanics.

## Methods

### Study population

HCHS/SOL is a community-based cohort study of 16,415 adults aged 18–74 yr who self-identified as Hispanic/Latino and were recruited in 2008–2011 from randomly selected households in 4 urban centers (Chicago, IL; Miami, FL; Bronx, NY; San Diego, CA). A two-stage area probability sample of households was selected with stratification and oversampling incorporated at each stage to provide a broadly diverse sample [20, 21]. These US communities are among the top eleven metropolitan areas with largest concentration of Hispanics/Latinos [22]. This study was approved by the coordinating center institutional review board along with the Internal Review Board of each respective field center and adults gave written informed consent. Data confidentiality was applied at all levels of data acquisition, transfer and storage.

**Fig. 1:**
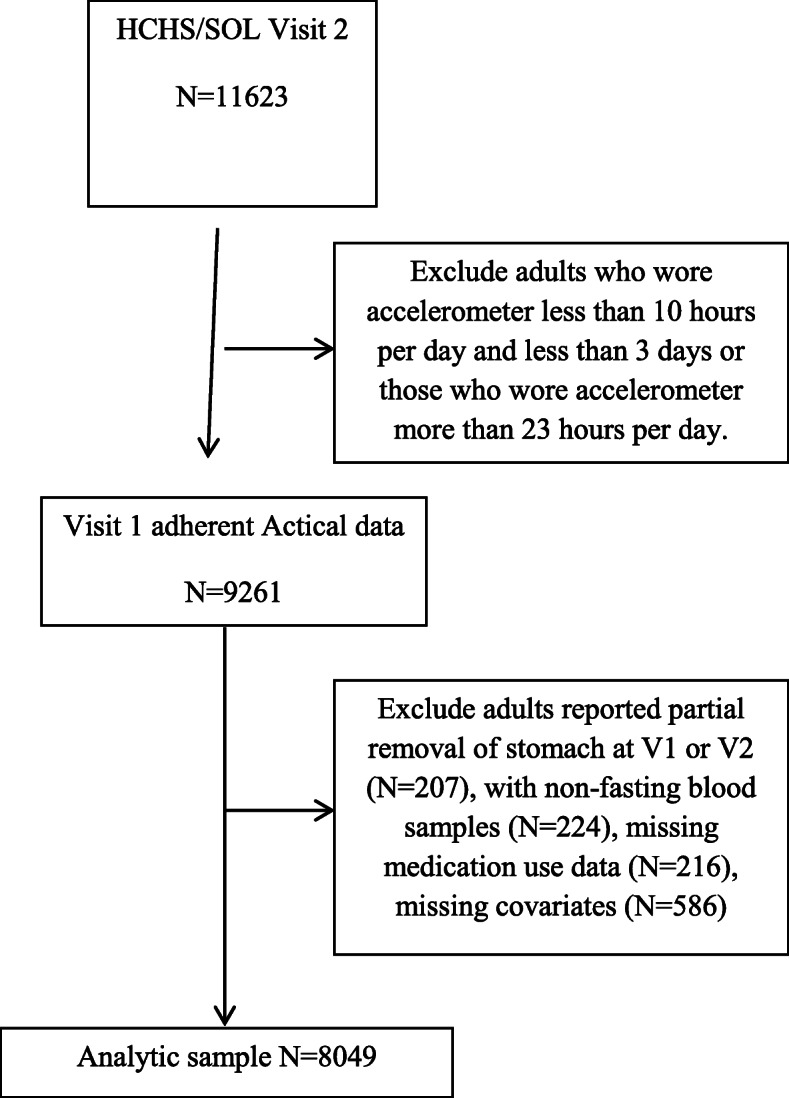
Participant Flow Chart

Participants had a comprehensive baseline Visit 1 (V1) examination that included biological, behavioral and socio-demographic assessments and were followed up yearly by telephone interview. Participants (~ 15,382 who have not moved out of the sampling area) were invited to a Visit 2 (V2) examination 6 years later and 11,623 participants completed the second assessment (2014–2017). In the current analysis 2362 participants were excluded who were not adherent to the accelerometry protocol. Non-adherence was defined as having fewer than 10 h per day and less than 3 days of data on the accelerometry or wearing the accelerometer for more than 23 h per day at V1. We further excluded participants who reported partial removal of the stomach at V1 or V2 (*N* = 207), or were missing a fasting blood sample (*N* = 224), medication use data related to blood pressure, lipid or glucose lowering (*N* = 216) or covariates (*N* = 586). Our final analytic sample consists of 8049 participants (Fig. 1).

Although HCHS/SOL has features of a population-based sample, we evaluated whether non-adherence to the accelerometry protocol may have produced a biased sample of participants who were included in these analyses. Adherence to accelerometry had been assessed previously for participants who had completed V1 [23]. Considering participants who attended V2, non-adherent participants were more likely to be younger, born in the US, (fifty states), have an education beyond high school, have a lower diet quality, and to smoke, and less likely to be employed full-time.

We did not find differences in cardiometabolic biomarkers based on adherence except that adherent participants were more likely to have pre-diabetes at V1 and/or have higher systolic blood pressure at both clinic visits and higher 2 h glucose at V2 compared to non-adherent adults (Additional Table 1).

**Table 1:** Age-adjusted baseline characteristics (95% CI) by quartiles of sedentary time and meeting guidelines for moderate-to-vigorous physical activity^a^

No. of adults	Sedentary time				*P*	MVPA guidelines^b^		*P*
	Quartile 1 (0.8–10.8 h)	Quartile 2 (10.8–12.0 h)	Quartile 3 (12.0–13.0 h)	Quartile 4 (13.0–16 h)		Not meet	Meet	
	2012	2012	2013	2012		4990	3059	
Sex, % male	57.2 (53.7, 60.5)	46.9 (43.3, 50.6)	44.2 (40.5, 48.1)	45.7 (42.2, 49.3)	< 0.0001	39.5 (37.1, 42.0)	59.8 (57.3, 62.3)	< 0.0001
Age in years, %					< 0.0001			< 0.0001
18–44	67.0 (64.0, 69.9)	65.7 (62.6, 68.6)	59.6 (56.2, 63.0)	53.0 (49.2, 56.7)		54.0 (51.6, 56.3)	70.5 (68.1, 72.7)	
45–64	30.6 (27.9, 33.5)	30.0 (27.3, 32.8)	31.5 (28.6, 34.5)	32.3 (29.6, 35.1)		35.2 (33.2, 37.1)	26.0 (24.0, 28.0)	
65–76	2.4 (1.6, 3.5)	4.3 (3.3, 5.7)	8.9 (7.1, 11.0)	14.7 (12.5, 17.2)		10.9 (9.4, 12.5)	3.6 (2.8, 4.6)	
Hispanic background, %					< 0.0001			< 0.0001
Dominican	6.4 (4.6, 8.9)	5.5 (4.1, 7.2)	7.9 (5.9, 10.4)	16.0 (13.3, 19.1)		6.1 (5.0, 7.4)	12.8 (10.6, 15.4)	
Central American	7.3 (5.6, 9.4)	7.9 (5.9, 10.5)	7.9 (6.3, 9.8)	8.8 (7.1, 11.0)		7.9 (6.5, 9.5)	8.0 (6.3, 10.2)	
Cuban	17.7 (13.9, 22.3)	20.9 (16.8, 25.7)	21.2 (17.3, 25.8)	19.1 (15.8, 22.9)		26.0 (22.1, 30.3)	11.5 (8.9, 14.7)	
Mexican	49.4 (44.2, 54.5)	41.4 (37.2, 45.8)	39.9 (35.3, 44.6)	27.7 (23.9, 31.8)		39.7 (35.8, 43.8)	39.5 (35.7, 43.4)	
Puerto Rican	10.5 (8.4, 13.2)	14.6 (12.1, 17.6)	14.5 (12.1, 17.3)	19.7 (16.9, 22.8)		12.5 (10.6, 14.6)	18.0 (15.9, 20.4)	
South American	4.1 (3.2, 5.4)	4.8 (3.5, 6.4)	5.1 (3.9, 6.6)	4.8 (3.5, 6.5)		4.2 (3.4, 5.2)	5.4 (4.4, 6.5)	
Other/> 1	4.5 (2.8, 7.2)	4.9 (2.9, 8.1)	3.5 (2.3, 5.3)	3.9 (2.7, 5.8)		3.6 (2.6, 5.0)	4.8 (3.6, 6.4)	
Born in 50 states, %	17.7 (14.9, 20.9)	21.8 (18.7, 25.3)	25.6 (22.5, 29.0)	25.7 (22.5, 29.3)	0.0003	20.9 (18.4, 23.7)	24.3 (21.8, 26.9)	0.05
≥ High school education, %	63.6 (59.8, 67.4)	69.2 (65.9, 72.4)	72.2 (69.1, 75.1)	67.9 (64.6, 71.0)	0.005	70.0 (67.7, 72.2)	65.9 (63.3, 68.5)	0.01
Annual family income, %					< 0.0001			0.86
< 20,000$	46.2 (42.1, 50.3)	44.0 (39.8, 48.2)	44.6 (40.5, 48.8)	49.9 (45.9, 53.9)		45.7 (42.6, 48.9)	46.8 (43.8, 49.9)	
20,000-50,000$	45.6 (41.8, 49.5)	42.6 (38.8, 46.4)	38.0 (34.4, 41.7)	38.3 (34.3, 42.4)		41.7 (38.8, 44.7)	40.7 (37.8, 43.7)	
> 50,000$	8.2 (6.4, 10.4)	13.5 (11.0, 16.4)	17.4 (14.1, 21.3)	11.8 (9.4, 14.7)		12.5 (10.5, 14.9)	12.4 (10.3, 15.0)	
Employed full/part-time, %	70.5 (67.1, 73.8)	54.4 (51.0, 57.7)	45.3 (41.7, 49.0)	38.5 (35.1, 42.0)	< 0.0001	50.3 (47.7, 52.9)	54.6 (51.8, 57.4)	0.02
Health insurance, %	43.0 (39.3, 46.8)	47.4 (43.6, 51.1)	51.7 (47.6, 55.8)	56.5 (52.6, 60.3)	< 0.0001	46.0 (43.4, 48.7)	53.9 (50.9, 56.9)	< 0.0001
Health care use, %	62.4 (58.7, 65.9)	66.4 (62.6, 70.0)	71.2 (67.8, 74.5)	72.6 (69.4, 75.6)	< 0.0001	67.4 (65.0, 69.7)	68.7 (66.0, 71.3)	0.44
Currently smokes, %	19.1 (16.4, 22.1)	18.7 (16.0, 21.8)	19.8 (16.8, 23.1)	17.4 (14.5, 20.8)	0.76	19.8 (17.5, 22.4)	17.5 (15.5, 19.8)	0.16
Alcohol use level, %					0.33			0.26
Non-drinker	46.5 (43.0, 50.2)	49.1 (44.9, 53.3)	49.9 (46.1, 53.7)	49.6 (46.0, 53.2)		48.2 (45.5, 50.9)	49.5 (46.7, 52.4)	
Moderate drinker	48.9 (45.3, 52.6)	44.1 (40.0, 48.4)	44.9 (41.0, 48.8)	45.4 (41.9, 49.0)		45.6 (42.9, 48.3)	46.0 (43.2, 48.8)	
Heavy drinker^c^	4.5 (3.4, 6.0)	6.8 (5.1, 8.9)	5.2 (3.6, 7.4)	5.0 (3.4, 7.2)		6.2 (4.8, 8.0)	4.5 (3.5, 5.8)	
Antidiabetic medication, %	6.7 (5.4, 8.4)	5.2 (4.2, 6.4)	7.8 (6.3, 9.6)	10.5 (9.1, 12.1)	< 0.0001	8.6 (7.6, 9.6)	6.4 (5.4, 7.6)	0.003
Antihypertensive medication, %	12.7 (11.1, 14.6)	14.2 (12.6, 16.0)	14.9 (13.1, 17.0)	18.8 (16.9, 20.9)	< 0.0001	16.7 (15.3, 18.2)	13.1 (11.7, 14.5)	< 0.0001
Lipid-lowering drugs, %	7.9 (6.6, 9.4)	7.3 (6.1, 8.7)	8.0 (6.7, 9.6)	10.3 (9.0, 11.9)	0.008	8.8 (7.8, 9.9)	8.2 (7.1, 9.4)	0.42
Alternative healthy eating index, mean	48.3 (47.8, 48.9)	48.1 (47.6, 48.6)	47.8 (47.2, 48.3)	46.7 (46.2, 47.2)	< 0.0001	47.4 (46.9, 47.8)	48.1 (47.7, 48.6)	0.002
Physical health score (SF-12), mean	51.1 (50.6, 51.6)	51.2 (50.7, 51.8)	49.9 (49.2, 50.5)	48.7 (48.0, 49.4)	< 0.0001	49.8 (49.3, 50.2)	50.8 (50.4, 51.2)	0.0002
BMI, kg/m^2^	28.8 (28.4, 29.3)	29.2 (28.8, 29.5)	29.1 (28.6, 29.7)	29.9 (29.4, 30.4)	0.01	29.8 (29.4, 30.2)	28.6 (28.3, 28.9)	< 0.0001
Waist-to-hip ratio	0.92 (0.91, 0.92)	0.92 (0.91, 0.92)	0.91 (0.91, 0.92)	0.92 (0.91, 0.92)	0.13	0.92 (0.92, 0.93)	0.91 (0.91, 0.91)	< 0.0001

^a^ Except for age, adjusted to the population-distribution by 10 year age groups as predicted marginals of the mean from linear regression for continuous variables or predicted marginals of the prevalence from logistic regression for categorical variables. ^b^ Meeting 2018 physical activity guidelines defined using measured activity scaled to 7 days of accelerometer wear as 150 minutes/week moderate intensity physical activity, 75 minutes/week vigorous intensity activity, or an equivalent combination of both. ^c^Heavy drinker was defined as 7 or more drinks/week for women and 14 or more drinks/week for men

### Physical activity and sedentary behavior assessment

At V1, all study participants were asked to wear an Actical accelerometer (version B-1; model 198–0200-03) for a week. Details of the accelerometer and assessment protocol are reported elsewhere [23, 24]. Wear time was identified using the Choi et al. algorithm [25]. The counts/min were used to categorize minutes in sedentary, light, moderate or vigorous activity by day, which were then averaged. Sedentary behavior was defined as < 100 cpm, light activity as 100–1534 cpm, moderate activity as 1535–3691 cpm, and vigorous activity as > 3961 cpm [26–28].

### Cardiometabolic biomarkers

Cardiometabolic biomarkers were measured at the V1 and V2 examinations. Based on a comparability study, biomarker results for V1 and V2 were comparable; coefficients of variation were 6–7% for all three liver enzymes: alanine amino transferase, aspartate amino transferase and gamma-glutamyl transferase [29]. Standing height was measured to the nearest centimeter. Body weight was measured using the Tanita scale to the nearest 0.1 kg and BMI was calculated as weight (kg) divided by height (m) squared. Obesity was defined as BMI ≥30 kg/m^2^. Waist circumference and hip circumference were measured to the nearest centimeter while adults stood erect, and waist-to-hip ratio was calculated. Participants were instructed to fast and refrain from smoking overnight for at least 8 h prior to the clinic visit. Sitting blood pressure was measured on the right arm three times with automatic sphygmomanometer (Omron HEM-907 XL) after participants rested for 5 min and then averaged. Blood samples were collected according to standardized protocol and quantified in the central laboratory. Details of methods used to quantify total cholesterol, HDL cholesterol, LDL cholesterol, triglycerides, fasting glucose, HbA1c, 2-h glucose (measured in non-diabetic adults), fasting insulin have been described elsewhere [17, 30]. HOMA-IR was calculated as fasting glucose (mg/dL) × fasting insulin (mU/L) /405 [31].

### Covariates

V1 covariates included age, sex, household income, education, employment status, nativity (born in 50 US States vs. not), Hispanic/Latino background, field center, smoking, alcohol consumption, health insurance status, health care use (defined as number of doctor visits in past year), self-reported health, diet quality as assessed by Alternative Healthy Eating Index (2010) (AHEI-2010), BMI and waist-to-hip ratio. Glucose, blood pressure, and lipid-lowering medication use was documented by directed questions at V1 and V2 six years later. We also included change in health insurance coverage from V1 to V2 (categorized as lost health insurance coverage, gained health insurance coverage and same status as V1).

### Statistical analyses

All analyses used complex survey methodology. We used sampling weights to account for V2 nonresponse and V1 oversampling of specific population subgroups. Weights were trimmed and calibrated to 2010 US Census characteristics by age, sex and Hispanic/Latino background in each field center’s target population.

To adjust for missing accelerometer data due to non-adherence to procedure (wore accelerometer < 10 h/day and < 3 days), we applied inverse probability weighting technique to all analyses. The probability weights of being adherent to Actical procedure at visit 1were calculated by fitting a logistic regression model with outcome as adherence (yes, no) and 16 predictors (details were described elsewhere) [17]. The weights used in the analyses were calculated as V2 sampling weight times inverse of the probability of being adherent to Actical procedure at V1.

Because of a high correlation between sedentary behavior and wear time, sedentary behavior was standardized to 16 h of wear time per day (the approximate average of both daily wear time and waking time in our study) using the residual from regressing sedentary behavior on wear time [32]. Unlike MVPA, for which there are guideline-driven criteria for desirable levels (e.g., “meets guidelines” or “does not meet guidelines”), sedentary behavior is not associated with guideline-specific cut-points. Therefore, we used the distribution of sedentary behavior within HCHS/SOL to derive categories for the sedentary behavior variable by quartiles, following our previously published work with these data [16, 17] [33]. MVPA was dichotomized as meeting or not meeting 2018 Physical Activity Guidelines [7] for adults. We also assessed MVPA continuously, but present the dichotomized version that reflected significant findings. Participants were considered to be meeting recommended activity levels if they had ≥75 weekly minutes of vigorous PA, or ≥ 150 weekly minutes of moderate PA, or an equivalent combination thereof. Light activity was assessed continuously (per hour).

We used age-adjusted means from survey linear regression or prevalence from survey logistic regression to summarize the distribution of participant characteristics such as sex, ethnicity, socio-economic status, health insurance, health care use, health-related behavior and medication use across quartiles of sedentary behavior and whether or not participants met MVPA guideline. To address the main study aim, we used survey linear regression models with changes in cardiometabolic markers as the dependent variables and quartiles of sedentary behavior or whether individuals met MVPA guidelines as the independent variables. Several cardiometabolic markers (e.g., triglycerides, fasting insulin and HOMA-IR) were natural log-transformed before analyses to normalize their distributions. For all models we excluded individuals with extreme changes in cardiometabolic markers defined as more than 3 standard deviations away from the mean.

Three nested models were fit. Model 1 was adjusted for age at V1, sex, use of medications that affects the dependent variable at V1 and/or V2, V1 levels of the dependent variables, and elapsed time between visits. Model 2 adjusted for Model 1 covariates and V1 household income, education, employment status, Hispanic/Latino background, field center, and nativity status, smoking, alcohol consumption, health insurance status, healthcare utilization, self-reported health, diet quality, change in health insurance, V1 BMI, and V1 waist-hip ratio (except in modeling change of BMI and waist circumference). Model 3 was adjusted for Model 2 covariates and time in sedentary behavior in models of MVPA or MVPA in models of time in sedentary behavior. P for trend was calculated using median time in sedentary behavior in each sedentary quartile in regression models. Analyses were conducted overall and excluding participants with cardiovascular disease and diabetes.

We adjusted for sociodemographic, behavioral and health–related confounders specific to each biomarker, using data from both V1 and V2 (blood –pressure lowering medications for blood pressure; lipid lowering drugs for blood lipids; glucose lowering medications for glycemic indices). We also stratified on pre-existing cardiometabolic disease. Cardiovascular disease was defined by electrocardiogram reports of possible past myocardial infarction, self-report of heart attack or procedure such as balloon angioplasty, stent, bypass surgery, self-report of stroke or mini-stroke or transient ischemic attack. Prediabetes was defined by fasting glucose 100–125 mg/dL, 2-h glucose 140–199 mg/dL, HbA1c 5.7–6.4% and not using antidiabetic diabetic medication. Diabetes was defined as fasting glucose ≥ 126 mg/dL, or 2-h glucose ≥ 200 mg/dL or HbA1c ≥6.5% or antidiabetic medication use. We also examined the associations of sedentary quartiles or MVPA with changes in cardiovascular biomarkers by age group (< 60 vs ≥60 years old), sex, Hispanic/Latino background and diabetes status at V1. Specifically, we included a product term of sedentary quartiles with these variables (i.e. age, sex) in the regression model and used F-test to identify significant interactions. The same was done for meeting or not meeting MVPA guidelines. We examined normoglycemic adults and adults with prediabetes both separately and combined. SAS and SUDAAN were used and all analyses were weighted and accounted for design effects.

## Results

Characteristics of the study population at V1 in lower vs. higher quartiles of sedentary behavior, and meeting vs. not meeting MVPA guidelines are indicated in Table 1. Sedentary behavior was lower in men, younger adults, adults employed part/full time. More women, older adults, and adults who were not employed (e.g. retirees, homemakers) had higher levels of sedentary behavior. Adults who were in lower quartile of sedentary behavior and also met the recommendations for MVPA guidelines took fewer glucose or blood pressure lowering medications, reported consuming a higher quality diet and had a lower BMI.

Overall we found no significant differences in change in cardiovascular biomarkers from V1 to V2 by quartile of sedentary behavior (Table 2). Adults who met MVPA guidelines at V1 had lower mean increases in fasting glucose, fasting insulin, and HOMA-IR than those who did not meet MVPA guidelines. Further adjusting for potential confounders attenuated the associations for fasting insulin and HOMA-IR, but not fasting glucose.

**Table 2:** Multivariable-adjusted mean changes in cardiovascular disease risk factors (95% CI) over 6 years of follow-up, according to quartiles of sedentary time and meeting guidelines for moderate-to-vigorous physical activity (N ~ 7900, 2 h glucose N ~ 5800)^a^ (No exclusions)

***Change in CVD risk factors***	Sedentary time				***P-trend***	MVPA guidelines^b^		***P***
	Quartile 1 (0.8–10.8 h)	Quartile 2 (10.8–12.0 h)	Quartile 3 (12.0–13.0 h)	Quartile 4 (13.0–16.0 h)		Not meet	Meet	
*BMI, kg/m2*								
*Model 1*	0.69 (0.50, 0.88)	0.83 (0.63, 1.04)	0.54 (0.34, 0.73)	0.68 (0.50, 0.87)	0.53	0.65 (0.53, 0.78)	0.73 (0.57, 0.89)	0.39
*Model 2*	0.67 (0.47, 0.87)	0.80 (0.60, 1.01)	0.55 (0.36, 0.74)	0.69 (0.50, 0.88)	0.75	0.62 (0.49, 0.74)	0.76 (0.60, 0.92)	0.16
*Model 3*	0.63 (0.41, 0.84)	0.80 (0.60, 1.00)	0.56 (0.37, 0.75)	0.73 (0.53, 0.92)	0.83	0.61 (0.47, 0.74)	0.77 (0.60, 0.93)	0.14
*Waist circumference, cm*								
*Model 1*	2.22 (1.72, 2.72)	2.23 (1.64, 2.81)	1.78 (1.16, 2.41)	2.96 (2.41, 3.52)	0.19	2.12 (1.74, 2.49)	2.53 (2.10, 2.96)	0.13
*Model 2*	2.36 (1.83, 2.89)	2.30 (1.71, 2.89)	1.84 (1.26, 2.43)	2.57 (2.01, 3.12)	0.97	2.25 (1.87, 2.63)	2.29 (1.87, 2.72)	0.88
*Model 3*	2.35 (1.78, 2.93)	2.30 (1.71, 2.89)	1.85 (1.26, 2.43)	2.57 (2.00, 3.15)	0.93	2.22 (1.82, 2.62)	2.33 (1.89, 2.77)	0.71
*Systolic BP, mmHg*								
*Model 1*	0.89 (0.14, 1.65)	1.39 (0.51, 2.27)	0.84 (0.01, 1.66)	0.69 (−0.13, 1.52)	0.60	0.94 (0.35, 1.52)	0.97 (0.31, 1.63)	0.94
*Model 2*	0.84 (0.02, 1.66)	1.45 (0.59, 2.30)	0.97 (0.15, 1.79)	0.59 (−0.24, 1.43)	0.64	1.04 (0.43, 1.65)	0.86 (0.21, 1.51)	0.68
*Model 3*	1.01 (0.13, 1.89)	1.45 (0.60, 2.30)	0.93 (0.10, 1.75)	0.46 (−0.38, 1.31)	0.34	1.05 (0.42, 1.68)	0.85 (0.15, 1.54)	0.69
*Diastolic BP, mmHg*								
*Model 1*	−0.17 (− 0.78, 0.45)	0.30 (− 0.32, 0.93)	−0.02 (− 0.66, 0.61)	0.26 (− 0.40, 0.93)	0.45	−0.14 (− 0.60, 0.32)	0.39 (− 0.12, 0.89)	0.115
*Model 2*	− 0.06 (− 0.69, 0.58)	0.47 (− 0.15, 1.08)	0.06 (− 0.55, 0.68)	−0.18 (− 0.85, 0.49)	0.73	0.01 (− 0.45, 0.47)	0.15 (− 0.36, 0.66)	0.67
*Model 3*	0.04 (− 0.65, 0.73)	0.48 (− 0.14, 1.09)	0.03 (− 0.59, 0.64)	−0.25 (− 0.92, 0.43)	0.48	0.04 (− 0.45, 0.52)	0.11 (− 0.44, 0.67)	0.84
*LDL-cholesterol, mg/dl*								
*Model 1*	−5.22 (−6.91, −3.53)	−3.89 (−5.97, −1.81)	−4.63 (− 6.64, −2.62)	−4.68 (− 6.70, − 2.67)	0.74	− 4.39 (− 5.74, − 3.03)	− 4.88 (− 6.32, − 3.44)	0.59
*Model 2*	−5.79 (−7.53, − 4.05)	−3.91 (− 6.03, − 1.79)	−4.25 (− 6.23, − 2.26)	−4.61 (− 6.48, − 2.74)	0.36	−4.21 (− 5.51, − 2.91)	− 5.18 (− 6.64, − 3.71)	0.28
*Model 3*	−5.94 (− 7.82, − 4.07)	−3.92 (− 6.05, − 1.79)	− 4.12 (− 6.13, − 2.12)	− 4.56 (− 6.46, − 2.67)	0.32	− 4.48 (− 5.83, − 3.12)	−4.85 (− 6.39, − 3.31)	0.71
*HDL-cholesterol, mg/dl*								
*Model 1*	1.27 (0.60, 1.94)	1.32 (0.72, 1.93)	2.17 (1.43, 2.90)	1.39 (0.76, 2.02)	0.42	1.61 (1.17, 2.06)	1.44 (0.93, 1.94)	0.61
*Model 2*	1.66 (0.94, 2.37)	1.34 (0.77, 1.91)	2.01 (1.31, 2.70)	1.12 (0.47, 1.77)	0.56	1.71 (1.25, 2.16)	1.31 (0.80, 1.82)	0.27
*Model 3*	1.80 (1.00, 2.59)	1.35 (0.79, 1.92)	1.99 (1.29, 2.70)	0.99 (0.30, 1.67)	0.34	1.69 (1.21, 2.17)	1.33 (0.78, 1.88)	0.37
*Triglycerides, mg/dl* ^*c*^								
*Model 1*	0.96 (0.92, 1.00)	0.93 (0.89, 0.96)	0.94 (0.91, 0.97)	0.95 (0.92, 0.98)	0.77	0.94 (0.92, 0.96)	0.95 (0.92, 0.97)	0.58
*Model 2*	0.94 (0.91, 0.98)	0.93 (0.89, 0.96)	0.94 (0.91, 0.97)	0.96 (0.93, 0.99)	0.58	0.94 (0.92, 0.96)	0.95 (0.93, 0.98)	0.30
*Model 3*	0.94 (0.90, 0.98)	0.93 (0.89, 0.96)	0.94 (0.91, 0.97)	0.96 (0.93, 1.00)	0.27	0.93 (0.91, 0.96)	0.95 (0.93, 0.98)	0.29
*Fasting glucose, mg/dl*								
*Model 1*	3.86 (2.94, 4.78)	4.26 (3.29, 5.22)	4.14 (3.08, 5.20)	4.33 (3.30, 5.35)	0.52	**4.84 (4.14, 5.54)**	**3.29 (2.54, 4.04)**	**0.001**
*Model 2*	3.47 (2.46, 4.48)	4.04 (3.08, 5.01)	4.34 (3.27, 5.41)	4.79 (3.76, 5.83)	0.072	**4.63 (3.94, 5.33)**	**3.58 (2.83, 4.34)**	**0.03**
*Model 3*	3.55 (2.46, 4.64)	4.09 (3.12, 5.06)	4.20 (3.12, 5.29)	4.81 (3.73, 5.88)	0.142	**4.73 (4.03, 5.44)**	**3.46 (2.64, 4.28)**	**0.01**
*2-h glucose, mg/dl*								
*Model 1*	7.57 (5.03, 10.10)	8.22 (5.59, 10.84)	7.06 (3.96, 10.16)	9.78 (7.21, 12.34)	0.37	8.17 (6.38, 9.96)	8.08 (6.04, 10.12)	0.94
*Model 2*	7.01 (4.48, 9.53)	8.21 (5.56, 10.85)	7.62 (4.63, 10.61)	9.86 (7.32, 12.39)	0.17	8.43 (6.72, 10.14)	7.80 (5.76, 9.83)	0.59
*Model 3*	7.48 (4.87, 10.09)	8.38 (5.72, 11.03)	7.23 (4.23, 10.23)	9.55 (6.82, 12.29)	0.43	8.29 (6.52, 10.06)	7.96 (5.87, 10.04)	0.79
*HbA1c, mg/dl*								
*Model 1*	0.18 (0.15, 0.21)	0.18 (0.15, 0.21)	0.15 (0.12, 0.18)	0.17 (0.14, 0.20)	0.28	0.16 (0.15, 0.18)	0.18 (0.15, 0.20)	0.28
*Model 2*	0.18 (0.15, 0.21)	0.18 (0.15, 0.21)	0.16 (0.13, 0.19)	0.17 (0.14, 0.20)	0.51	0.17 (0.15, 0.18)	0.18 (0.16, 0.20)	0.35
*Model 3*	0.18 (0.15, 0.21)	0.18 (0.15, 0.21)	0.16 (0.13, 0.19)	0.17 (0.14, 0.20)	0.69	0.17 (0.15, 0.19)	0.17 (0.15, 0.20)	0.98
*Fasting insulin, mU/L* ^*c*^								
*Model 1*	1.20 (1.15, 1.25)	1.23 (1.18, 1.30)	1.19 (1.14, 1.24)	1.21 (1.16, 1.25)	0.82	**1.23 (1.20, 1.27)**	**1.18 (1.14, 1.22)**	**0.02**
*Model 2*	1.18 (1.13, 1.23)	1.22 (1.17, 1.28)	1.20 (1.15, 1.25)	1.23 (1.19, 1.28)	0.13	1.22 (1.19, 1.26)	1.19 (1.14, 1.23)	0.10
*Model 3*	1.19 (1.13, 1.24)	1.23 (1.17, 1.29)	1.19 (1.14, 1.24)	1.22 (1.18, 1.27)	0.55	1.23 (1.19, 1.26)	1.18 (1.14, 1.23)	0.06
*HOMA-IR* ^*c*^								
*Model 1*	1.27 (1.21, 1.32)	1.29 (1.22, 1.36)	1.24 (1.18, 1.30)	1.25 (1.20, 1.31)	0.55	**1.29 (1.25, 1.33)**	**1.23 (1.18, 1.27)**	**0.02**
*Model 2*	1.23 (1.17, 1.28)	1.27 (1.21, 1.34)	1.25 (1.19, 1.31)	1.29 (1.24, 1.34)	0.13	1.28 (1.24, 1.32)	1.24 (1.19, 1.29)	0.13
*Model 3*	1.24 (1.18, 1.31)	1.28 (1.21, 1.35)	1.24 (1.18, 1.30)	1.28 (1.23, 1.33)	0.55	1.28 (1.24, 1.32)	1.23 (1.18, 1.28)	0.07

^a^Extreme values of change in biomarker (>3SD of distribution) were excluded; extreme observations differ according to different biomarkers- so sample size is different for analysis of each biomarker

Model 1 adjusted for age at baseline, sex, use of medications that affect the dependent variable at baseline and/or visit2, baseline levels of the dependent variable, and elapsed time between visits

Model 2 further adjusted for baseline household income, education, employment status, Hispanic/Latino background, field center, and nativity status, smoking, alcohol consumption, health insurance status, healthcare utilization, self-reported health, diet quality, change in health insurance, baseline BMI and waist-hip ratio (except in modeling change of BMI and waist circumference)

Model 3 adjusted for Model 2 covariates and sedentary time in models of MVPA or MVPA in models of sedentary time

^b^Meeting 2018 physical activity guidelines defined using measured activity scaled to 7 days of accelerometer wear as 150 minutes/week moderate intensity physical activity, 75 minutes/week vigorous intensity activity, or an equivalent combination of both

^c^Geometric means (95% CI) presented for triglycerides, fasting insulin, HOMA-IR

Significant findings *p* < 0.05 bolded

Normoglycemic adults without cardiovascular disease or diabetes in the lowest quartile for sedentary behavior had significantly more of a decrease in LDL-cholesterol of − 3.94 mg/dL (95% CI: − 6.37, − 1.52) compared to the highest quartile: 0.14 mg/dL (95% CI: − 2.15, 2.42) in fully adjusted models (Table 3). Meeting MVPA guidelines was associated with less of an increase in level of fasting glucose and HOMA-IR in adjusted models. Larger decrease in mean LDL-cholesterol and lower mean increase in HbA1c was associated with being in the lower as compared to the higher sedentary quartiles.

**Table 3:** Multivariable-adjusted mean changes in cardiovascular disease risk factors (95% CI) over 6 years of follow-up among individuals without CVD or diabetes or prediabetes at baseline, (normoglycemic adults only) according to quartiles of sedentary time and meeting guidelines for moderate-to-vigorous physical activity (N ~ 3014)

Change in CVD risk factors	Sedentary time				***P-trend***	MVPA guidelines^a^		***P***
	Quartile 1 (3.4–10.7 h)	Quartile 2 (10.7–11.9 h)	Quartile 3 (11.9–12.8 h)	Quartile 4 (12.8–16.0 h)		Not meet	Meet	
BMI, kg/m2								
Model 1	1.17 (0.86, 1.49)	1.22 (0.95, 1.48)	1.17 (0.83, 1.51)	1.31 (1.06, 1.56)	0.58	1.14 (0.95, 1.34)	1.29 (1.08, 1.49)	0.30
Model 2	1.16 (0.83, 1.49)	1.19 (0.94, 1.44)	1.16 (0.85, 1.47)	1.31 (1.05, 1.57)	0.51	1.08 (0.89, 1.28)	1.33 (1.11, 1.54)	0.09
Model 3	1.10 (0.75, 1.46)	1.19 (0.93, 1.44)	1.18 (0.86, 1.49)	1.35 (1.08, 1.62)	0.32	**1.04 (0.83, 1.25)**	**1.37 (1.15, 1.60)**	**0.04**
Waist circumference, cm								
Model 1	2.83 (1.97, 3.69)	3.03 (2.16, 3.90)	2.86 (1.83, 3.90)	3.80 (3.02, 4.59)	0.17	**2.67 (2.02, 3.31)**	**3.62 (3.03, 4.20)**	**0.02**
Model 2	2.91 (1.97, 3.85)	3.09 (2.26, 3.92)	2.97 (2.05, 3.89)	3.38 (2.61, 4.15)	0.51	2.83 (2.19, 3.46)	3.36 (2.75, 3.96)	0.20
Model 3	2.75 (1.71, 3.80)	3.08 (2.25, 3.91)	3.01 (2.09, 3.94)	3.49 (2.69, 4.30)	0.35	2.73 (2.06, 3.39)	3.46 (2.83, 4.09)	0.11
Systolic BP, mmHg								
Model 1	1.37 (0.20, 2.54)	0.51 (−0.82, 1.85)	1.09 (−0.06, 2.24)	0.60 (− 0.52, 1.72)	0.42	1.00 (0.10, 1.89)	0.76 (− 0.20, 1.72)	0.73
Model 2	1.40 (0.15, 2.65)	0.64 (−0.67, 1.95)	1.44 (0.30, 2.57)	0.37 (−0.74, 1.48)	0.34	1.01 (0.11, 1.91)	0.86 (−0.12, 1.84)	0.83
Model 3	1.69 (0.38, 3.01)	0.63 (−0.67, 1.94)	1.31 (0.16, 2.46)	0.23 (−0.88, 1.34)	0.16	1.06 (0.11, 2.00)	0.81 (−0.20, 1.83)	0.75
Diastolic BP, mmHg								
Model 1	1.52 (0.46, 2.58)	1.26 (0.32, 2.20)	1.27 (0.24, 2.29)	1.78 (0.84, 2.71)	0.78	1.28 (0.54, 2.01)	1.62 (0.87, 2.36)	0.52
Model 2	1.75 (0.65, 2.85)	1.47 (0.52, 2.42)	1.35 (0.39, 2.30)	1.32 (0.44, 2.20)	0.53	1.39 (0.68, 2.10)	1.51 (0.73, 2.28)	0.83
Model 3	1.98 (0.77, 3.19)	1.48 (0.52, 2.43)	1.29 (0.31, 2.27)	1.16 (0.26, 2.06)	0.30	1.47 (0.69, 2.25)	1.43 (0.62, 2.23)	0.94
LDL-cholesterol, mg/dl								
Model 1	**−3.33 (−5.59, −1.07)**	**−0.35 (−3.09, 2.38)**	**−0.41 (−3.21, 2.39)**	**−0.07 (−2.58, 2.43)**	**0.05**	− 0.05 (−2.14, 2.05)	−2.06 (− 3.81, − 0.31)	0.13
Model 2	**−4.05 (−6.29, − 1.80)**	**−0.34 (− 2.93, 2.25)**	**−0.17 (− 2.75, 2.41)**	**0.16 (− 2.06, 2.39)**	**0.01**	0.21 (−1.69, 2.10)	−2.40 (− 4.21, − 0.59)	0.04
Model 3	**− 3.94 (− 6.37, − 1.52)**	**− 0.32 (− 2.94, 2.31)**	**−0.27 (− 2.84, 2.31)**	**0.14 (−2.15, 2.42)**	**0.03**	− 0.60 (− 2.54, 1.33)	− 1.61 (− 3.52, 0.30)	0.47
HDL-cholesterol, mg/dl								
Model 1	1.13 (0.04, 2.23)	0.95 (0.02, 1.89)	2.36 (1.06, 3.66)	1.31 (0.33, 2.29)	0.49	1.69 (0.94, 2.44)	1.22 (0.49, 1.96)	0.39
Model 2	1.66 (0.45, 2.86)	0.90 (0.06, 1.74)	2.19 (1.00, 3.39)	0.94 (−0.08, 1.96)	0.66	1.77 (1.00, 2.55)	1.09 (0.37, 1.82)	0.21
Model 3	1.92 (0.54, 3.29)	0.92 (0.08, 1.76)	2.13 (0.92, 3.35)	0.74 (−0.33, 1.81)	0.41	1.80 (0.98, 2.62)	1.06 (0.27, 1.85)	0.23
Triglycerides, mg/dl ^b^								
Model 1	1.02 (0.97, 1.08)	0.96 (0.90, 1.02)	0.96 (0.92, 1.01)	1.00 (0.95, 1.05)	0.48	0.98 (0.96, 1.01)	0.99 (0.95, 1.03)	0.92
Model 2	1.01 (0.95, 1.07)	0.96 (0.91, 1.02)	0.96 (0.92, 1.01)	1.00 (0.96, 1.05)	0.73	0.97 (0.94, 1.00)	0.99 (0.96, 1.03)	0.38
Model 3	1.00 (0.94, 1.06)	0.96 (0.91, 1.02)	0.97 (0.92, 1.01)	1.01 (0.97, 1.06)	0.77	0.97 (0.94, 1.01)	0.99 (0.95, 1.03)	0.47
Fasting glucose, mg/dl								
Model 1	3.82 (2.99, 4.64)	3.12 (2.28, 3.95)	3.64 (2.79, 4.49)	3.79 (2.90, 4.68)	0.94	**4.28 (3.67, 4.89)**	**2.89 (2.30, 3.49)**	**0.0004**
Model 2	3.41 (2.58, 4.24)	3.04 (2.26, 3.82)	3.64 (2.80, 4.47)	4.08 (3.23, 4.93)	0.22	**3.94 (3.33, 4.54)**	**3.16 (2.57, 3.75)**	**0.05**
Model 3	3.38 (2.52, 4.24)	3.04 (2.26, 3.82)	3.67 (2.83, 4.51)	4.08 (3.21, 4.94)	0.21	**4.00 (3.40, 4.60)**	**3.10 (2.49, 3.71)**	**0.02**
2-h glucose, mg/dl								
Model 1	10.78 (7.52, 14.04)	8.92 (5.17, 12.66)	8.66 (4.79, 12.53)	12.47 (9.49, 15.44)	0.56	10.23 (7.81, 12.66)	10.06 (7.38, 12.75)	0.92
Model 2	10.08 (6.82, 13.34)	9.14 (5.36, 12.92)	8.51 (4.86, 12.16)	13.11 (10.21, 16.00)	0.22	10.15 (7.86, 12.44)	10.17 (7.42, 12.92)	0.99
Model 3	11.84 (8.62, 15.06)	9.20 (5.44, 12.96)	8.07 (4.27, 11.87)	11.83 (8.82, 14.84)	0.77	9.70 (7.33, 12.07)	10.61 (7.89, 13.33)	0.58
HbA1c, mg/dl								
Model 1	**0.13 (0.11, 0.15)**	**0.14 (0.12, 0.16)**	**0.16 (0.13, 0.18)**	**0.17 (0.14, 0.19)**	**0.02**	0.15 (0.13, 0.16)	0.15 (0.13, 0.17)	0.64
Model 2	**0.13 (0.10, 0.15)**	**0.14 (0.12, 0.16)**	**0.16 (0.14, 0.19)**	**0.16 (0.14, 0.19)**	**0.02**	0.15 (0.13, 0.16)	0.15 (0.13, 0.17)	0.68
Model 3	0.13 (0.11, 0.16)	0.14 (0.12, 0.16)	0.16 (0.14, 0.19)	0.16 (0.14, 0.19)	0.08	0.14 (0.13, 0.16)	0.16 (0.14, 0.17)	0.24
Fasting insulin, mU/L ^b^								
Model 1	1.28 (1.20, 1.38)	1.25 (1.17, 1.34)	1.29 (1.21, 1.38)	1.28 (1.21, 1.36)	0.97	**1.32 (1.26, 1.37)**	**1.24 (1.18, 1.30)**	**0.02**
Model 2	1.24 (1.16, 1.33)	1.25 (1.18, 1.33)	1.28 (1.21, 1.37)	1.33 (1.25, 1.41)	0.09	1.30 (1.25, 1.36)	1.25 (1.19, 1.31)	0.14
Model 3	1.26 (1.17, 1.35)	1.25 (1.18, 1.33)	1.29 (1.21, 1.38)	1.30 (1.23, 1.38)	0.36	1.30 (1.25, 1.36)	1.25 (1.18, 1.31)	0.11
HOMA-IR^b^								
Model 1	1.34 (1.24, 1.44)	1.29 (1.20, 1.38)	1.34 (1.25, 1.44)	1.33 (1.25, 1.42)	0.94	**1.38 (1.31, 1.44)**	**1.27 (1.21, 1.34)**	**0.005**
Model 2	1.28 (1.19, 1.38)	1.28 (1.20, 1.37)	1.33 (1.25, 1.43)	1.39 (1.31, 1.48)	0.07	**1.36 (1.30, 1.42)**	**1.29 (1.22, 1.36)**	**0.07**
Model 3	1.30 (1.20, 1.41)	1.29 (1.21, 1.37)	1.34 (1.25, 1.44)	1.36 (1.28, 1.45)	0.30	1.36 (1.30, 1.43)	1.28 (1.22, 1.35)	0.05

Excluded 5035 adults with prediabetes (*n* = 3301), diabetes (*n* = 1629) or CVD (*n* = 509). Note adults may have prediabetes with CVD (*n* = 201), or diabetes with CVD (*n* = 203).

^a^Meeting 2018 physical activity guidelines defined using measured activity scaled to 7 days of accelerometer wear as 150 min/week moderate-intensity physical activity, 75 min/week vigorous-intensity physical activity, or an equivalent combination of both

^b^Geometric means (95% CI) presented for triglycerides, fasting insulin, HOMA-IR

Model 1 adjusted for age at baseline, sex, use of medications that affect the dependent variable at baseline and/or visit2, baseline levels of the dependent variable, and elapsed time between visits

Model 2 further adjusted for baseline household income, education, employment status, Hispanic/Latino background, field center, and nativity status, smoking, alcohol consumption, health insurance status, healthcare utilization, self-reported health, diet quality and change in health insurance

Model 3 adjusted for Model 2 covariates and sedentary time in models of MVPA or MVPA in models of sedentary time

Significant findings p < 0.05 bolded

Individuals with prediabetes and individuals with normal glycemic levels (Table 4) who met MVPA guidelines had significantly lower increases in fasting glucose in the fully adjusted model than adults not meeting MVPA guidelines. An additional analysis (Additional Table 2) which only included individuals with prediabetes at V1 (that excluded adults with diabetes at V2) did not indicate any changes in CVD biomarkers over six years regardless of sedentary behavior. Likewise, no changes were observed with adults with prediabetes at V1 meeting MVPA compared to adults not meeting MVPA recommendations.

**Table 4:** Multivariable-adjusted mean changes in cardiovascular disease risk factors (95% CI) over 6 years of follow-up among individuals without CVD or diabetes (includes normoglycemic adults and adults with prediabetes), according to quartiles of sedentary time and meeting guidelines for moderate-to-vigorous physical activity (N ~ 6114)

Change in CVD risk factors	Sedentary time				***P-trend***	MVPA guidelines^a^		***P***
	Quartile 1 (0.8–10.8 h)	Quartile 2 (10.8–11.9 h)	Quartile 3 (11.9–12.8 h)	Quartile 4 (12.8–16.0 h)		Not meet	Meet	
BMI, kg/m2								
Model 1	0.90 (0.68, 1.13)	1.06 (0.84, 1.28)	0.83 (0.61, 1.06)	0.93 (0.74, 1.12)	0.86	0.89 (0.75, 1.03)	0.98 (0.81, 1.16)	0.35
Model 2	0.88 (0.65, 1.11)	1.04 (0.82, 1.26)	0.84 (0.62, 1.06)	0.93 (0.74, 1.13)	0.96	0.85 (0.71, 0.99)	1.01 (0.83, 1.18)	0.14
Model 3	0.83 (0.58, 1.07)	1.04 (0.82, 1.26)	0.85 (0.64, 1.07)	0.98 (0.78, 1.18)	0.56	0.83 (0.68, 0.98)	1.03 (0.86, 1.20)	0.09
Waist circumference, cm								
Model 1	2.55 (1.97, 3.14)	2.70 (2.01, 3.38)	2.46 (1.75, 3.18)	3.23 (2.65, 3.81)	0.22	2.49 (2.05, 2.93)	3.02 (2.55, 3.49)	0.08
Model 2	2.69 (2.06, 3.31)	2.76 (2.08, 3.44)	2.53 (1.86, 3.20)	2.86 (2.27, 3.45)	0.84	2.66 (2.21, 3.11)	2.77 (2.31, 3.24)	0.70
Model 3	2.64 (1.96, 3.31)	2.76 (2.08, 3.44)	2.54 (1.87, 3.22)	2.90 (2.29, 3.51)	0.72	2.62 (2.15, 3.08)	2.81 (2.34, 3.29)	0.55
Systolic BP, mmHg								
Model 1	0.69 (−0.15, 1.52)	0.97 (0.04, 1.90)	0.46 (− 0.44, 1.36)	0.56 (− 0.28, 1.41)	0.69	0.70 (0.06, 1.35)	0.63 (− 0.09, 1.34)	0.88
Model 2	0.70 (−0.20, 1.61)	0.99 (0.09, 1.88)	0.62 (− 0.28, 1.52)	0.42 (− 0.44, 1.28)	0.61	0.77 (0.10, 1.43)	0.58 (− 0.14, 1.30)	0.71
Model 3	0.87 (−0.10, 1.83)	0.99 (0.10, 1.89)	0.58 (−0.33, 1.48)	0.30 (− 0.56, 1.17)	0.36	0.78 (0.09, 1.47)	0.56 (− 0.20, 1.32)	0.69
Diastolic BP, mmHg								
Model 1	0.14 (−0.57, 0.85)	0.58 (−0.08, 1.25)	0.19 (− 0.54, 0.92)	0.83 (0.11, 1.54)	0.27	0.24 (− 0.28, 0.76)	0.66 (0.10, 1.21)	0.27
Model 2	0.30 (−0.44, 1.04)	0.70 (0.04, 1.37)	0.26 (−0.45, 0.97)	0.42 (− 0.27, 1.11)	0.96	0.43 (− 0.10, 0.95)	0.41 (− 0.16, 0.98)	0.97
Model 3	0.44 (−0.37, 1.24)	0.72 (0.05, 1.38)	0.21 (− 0.50, 0.93)	0.32 (− 0.38, 1.02)	0.67	0.44 (− 0.12, 0.99)	0.40 (− 0.21, 1.01)	0.93
LDL-cholesterol, mg/dl								
Model 1	−4.13 (−5.90, −2.35)	−2.58 (−5.00, − 0.17)	−3.45 (− 5.53, −1.37)	−3.24 (− 5.18, −1.31)	0.61	−2.95 (− 4.38, − 1.52)	−3.80 (− 5.35, − 2.24)	0.40
Model 2	−4.52 (−6.40, − 2.65)	− 2.64 (− 5.04, − 0.23)	− 3.07 (− 5.11, − 1.03)	−3.32 (− 5.10, − 1.55)	0.41	− 2.66 (− 4.08, − 1.25)	− 4.19 (− 5.75, − 2.63)	0.13
Model 3	−4.71 (− 6.73, − 2.69)	−2.68 (− 5.10, − 0.25)	−2.96 (− 5.02, − 0.90)	− 3.21 (− 5.04, − 1.38)	0.35	−2.96 (− 4.44, − 1.48)	−3.87 (− 5.50, − 2.24)	0.41
HDL-cholesterol, mg/dl								
Model 1	1.22 (0.46, 1.98)	1.19 (0.48, 1.89)	2.11 (1.23, 3.00)	1.60 (0.91, 2.29)	0.26	1.68 (1.17, 2.19)	1.37 (0.82, 1.93)	0.43
Model 2	1.66 (0.85, 2.48)	1.22 (0.57, 1.88)	1.94 (1.10, 2.77)	1.28 (0.56, 1.99)	0.75	1.74 (1.21, 2.26)	1.29 (0.73, 1.85)	0.27
Model 3	1.86 (0.95, 2.77)	1.23 (0.57, 1.88)	1.91 (1.06, 2.76)	1.11 (0.36, 1.86)	0.43	1.71 (1.15, 2.27)	1.32 (0.71, 1.93)	0.39
Triglycerides, mg/dl^b^								
Model 1	0.97 (0.93, 1.01)	0.95 (0.91, 0.99)	0.95 (0.91, 0.98)	0.97 (0.94, 1.00)	0.88	0.96 (0.93, 0.98)	0.96 (0.93, 0.99)	0.93
Model 2	0.95 (0.91, 0.99)	0.95 (0.91, 0.99)	0.95 (0.92, 0.99)	0.97 (0.94, 1.00)	0.51	0.95 (0.93, 0.98)	0.96 (0.93, 0.99)	0.62
Model 3	0.94 (0.90, 0.99)	0.95 (0.91, 0.98)	0.96 (0.92, 0.99)	0.98 (0.95, 1.01)	0.27	0.95 (0.93, 0.98)	0.96 (0.93, 0.99)	0.59
Fasting glucose, mg/dl								
Model 1	3.46 (2.73, 4.19)	4.45 (3.52, 5.39)	3.53 (2.78, 4.27)	3.87 (3.11, 4.63)	0.71	**4.60 (4.04, 5.17)**	**2.96 (2.36, 3.56)**	**< 0.0001**
Model 2	2.92 (2.15, 3.69)	4.37 (3.45, 5.29)	3.69 (2.94, 4.44)	4.25 (3.48, 5.02)	0.04	**4.23 (3.67, 4.80)**	**3.34 (2.75, 3.93)**	**0.01**
Model 3	2.92 (2.10, 3.74)	4.38 (3.46, 5.31)	3.63 (2.88, 4.39)	4.29 (3.49, 5.10)	0.07	**4.32 (3.73, 4.91)**	**3.25 (2.61, 3.89)**	**0.01**
2-h glucose, mg/dl								
Model 1	7.75 (5.17, 10.32)	9.70 (6.93, 12.48)	8.11 (4.88, 11.34)	9.48 (7.14, 11.82)	0.44	8.55 (6.74, 10.36)	8.99 (6.88, 11.10)	0.73
Model 2	6.92 (4.37, 9.47)	9.93 (7.08, 12.78)	8.48 (5.34, 11.61)	9.75 (7.40, 12.11)	0.14	8.78 (7.00, 10.55)	8.77 (6.70, 10.84)	0.99
Model 3	7.36 (4.73, 9.98)	10.04 (7.19, 12.89)	8.25 (5.07, 11.43)	9.45 (6.90, 12.00)	0.39	8.63 (6.78, 10.48)	8.93 (6.81, 11.05)	0.82
HbA1c, mg/dl								
Model 1	0.15 (0.13, 0.18)	0.18 (0.16, 0.21)	0.15 (0.13, 0.17)	0.17 (0.15, 0.19)	0.63	0.16 (0.14, 0.17)	0.17 (0.15, 0.19)	0.25
Model 2	0.15 (0.13, 0.17)	0.18 (0.16, 0.21)	0.16 (0.14, 0.18)	0.17 (0.15, 0.19)	0.21	0.16 (0.14, 0.17)	0.17 (0.15, 0.19)	0.18
Model 3	0.14 (0.12, 0.16)	0.18 (0.16, 0.21)	0.16 (0.14, 0.18)	0.17 (0.15, 0.20)	0.11	0.16 (0.14, 0.17)	0.17 (0.15, 0.19)	0.33
Fasting insulin, mU/L^b^								
Model 1	1.23 (1.17, 1.29)	1.28 (1.21, 1.35)	1.23 (1.17, 1.29)	1.24 (1.18, 1.29)	0.99	1.27 (1.23, 1.31)	1.21 (1.17, 1.26)	0.03
Model 2	1.19 (1.14, 1.25)	1.26 (1.20, 1.33)	1.24 (1.18, 1.30)	1.27 (1.22, 1.33)	0.05	1.26 (1.22, 1.30)	1.22 (1.18, 1.27)	0.16
Model 3	1.20 (1.14, 1.27)	1.27 (1.20, 1.34)	1.23 (1.18, 1.30)	1.26 (1.21, 1.31)	0.28	1.26 (1.22, 1.31)	1.22 (1.17, 1.27)	0.10
HOMA-IR^b^								
Model 1	1.28 (1.22, 1.35)	1.33 (1.25, 1.41)	1.27 (1.21, 1.34)	1.29 (1.23, 1.35)	0.85	1.33 (1.28, 1.37)	1.25 (1.20, 1.31)	0.01
Model 2	1.23 (1.17, 1.30)	1.31 (1.24, 1.39)	1.29 (1.22, 1.36)	1.33 (1.27, 1.39)	0.04	1.31 (1.27, 1.36)	1.27 (1.22, 1.32)	0.11
Model 3	1.25 (1.18, 1.32)	1.32 (1.24, 1.40)	1.28 (1.22, 1.35)	1.32 (1.26, 1.38)	0.21	1.32 (1.27, 1.37)	1.26 (1.21, 1.32)	0.06

Excluded 1935 adults with diabetes (n = 1629) or CVD (n = 509). Note 203 adults had both diabetes and CVD

Model 2 further adjusted for baseline household income, education, employment status, Hispanic/Latino background, field center, and nativity status, smoking, alcohol consumption, health insurance status, healthcare utilization, self-reported health, diet quality, change in health insurance, baseline BMI and waist-hip ratio (except in modeling change of BMI and waist circumference)

Model 3 adjusted for Model 2 covariates and sedentary time in models of MVPA or MVPA in models of sedentary time

^a^Meeting 2018 physical activity guidelines defined using measured activity scaled to 7 days of accelerometer wear as 150 minutes/week moderate intensityphysical activity, 75 minutes/week vigorous intensity activity, or an equivalent combination of both

^b^Geometric means (95% CI) presented for triglycerides, fasting insulin, HOMA-IR

Light activity was highly inversely correlated with sedentary time with correlation coefficient of − 0.91 and was associated with decrease in LDL cholesterol over six years in adults without cardiovascular disease or prediabetes. The estimate in change in LDL cholesterol (mg/dL) by light activity (per hour) was: Model 1: -0.92 (95% CI: − 1.62, − 0.16), *P* = 0.02; Model 2: -1.06 (95% CI: − 1.84, − 0.28), *P* = 0.008 (Data not shown).

LDL cholesterol was the only biomarker that decreased from V1 to V2 among adults with normoglycemia or prediabetes. While the decrease was greatest in adults on statins at V2 and not at V1 (mean decrease of − 35.6 (36.3) mg/dL in *n* = 898 adults), even adults who were not on statins at both visits experienced a mean decrease in LDL cholesterol of − 3.8 (23.3) mg/dL (Additional Table 3). We explored whether the association between MVPA and cardiometabolic outcomes differed across subgroups of the population. We found no significant effect modification by age (< 60 vs ≥60 years old) and diabetes status at V1 for all adults on the association of sedentary behavior and MVPA with changes in cardiometabolic biomarkers across six years (data not shown).

Effect modification by sex was only found in the associations between meeting MVPA guidelines and changes in both fasting insulin ((P for interaction = 0.01) and HOMA-IR (P for interaction = 0.01) Table 5.) Sex stratified analyses showed that women meeting MVPA guidelines experienced reductions over time in both biomarkers (fasting insulin & HOMA-IR). For men there was no association between meeting MVPA guidelines and these cardiometabolic biomarkers.

**Table 5:** Multivariable-adjusted mean changes in cardiovascular disease risk factors (95% CI) over 6 years of follow-up, according to quartiles of sedentary time and meeting guidelines for moderate-to-vigorous physical activity among men and women – No exclusions^a^

Change in CVD risk factors	Sedentary time				P of inter-action	MVPA guidelines^b^		P of inter-action
	Quartile 1 (0.8–10.8 h)	Quartile 2 (10.8–12.0 h)	Quartile 3 (12.0–13.0 h)	Quartile 4 (13.0–16.0 h)		Not meet	Meet	
BMI, kg/m2					0.59			0.99
Women	0.68 (0.40, 0.95)	0.85 (0.63, 1.08)	0.74 (0.50, 0.98)	0.83 (0.56, 1.09)		0.73 (0.57, 0.90)	0.86 (0.65, 1.07)	
Men	0.60 (0.33, 0.87)	0.75 (0.42, 1.08)	0.32 (0.04, 0.60)	0.61 (0.33, 0.89)		0.51 (0.31, 0.70)	0.63 (0.40, 0.86)	
Waist circumference, cm					0.08			0.79
Women	1.52 (0.76, 2.28)	1.64 (0.88, 2.40)	1.92 (1.27, 2.57)	2.26 (1.49, 3.03)		1.87 (1.36, 2.38)	1.84 (1.30, 2.37)	
Men	3.12 (2.37, 3.88)	3.03 (2.16, 3.90)	1.64 (0.68, 2.59)	2.88 (2.02, 3.73)		2.64 (2.07, 3.22)	2.75 (2.09, 3.41)	
Systolic BP, mmHg					0.43			0.67
Women	−0.00 (−1.10, 1.09)	0.96 (−0.14, 2.05)	0.50 (− 0.67, 1.68)	0.58 (− 0.54, 1.70)		0.51 (− 0.22, 1.24)	0.60 (− 0.37, 1.57)	
Men	1.77 (0.59, 2.94)	1.88 (0.62, 3.13)	1.41 (0.08, 2.75)	0.48 (− 0.76, 1.72)		1.55 (0.46, 2.64)	1.28 (0.50, 2.06)	
Diastolic BP, mmHg					0.72			0.99
Women	−0.49 (−1.36, 0.38)	0.40 (−0.38, 1.18)	− 0.21 (− 0.98, 0.55)	−0.48 (− 1.34, 0.38)		− 0.25 (− 0.79, 0.29)	−0.04 (− 0.79, 0.72)	
Men	0.58 (−0.31, 1.48)	0.64 (− 0.31, 1.59)	0.32 (− 0.76, 1.39)	−0.22 (− 1.24, 0.81)		0.22 (− 0.56, 1.00)	0.42 (− 0.24, 1.08)	
LDL-cholesterol, mg/dl					0.34			0.15
Women	−4.62 (−6.73, −2.51)	−4.48 (−6.54, −2.41)	−3.63 (−5.97, − 1.30)	−5.39 (−7.57, −3.20)		−3.74 (− 5.34, − 2.13)	−6.01 (− 7.96, − 4.05)	
Men	− 6.38 (−8.88, − 3.89)	−3.18 (− 6.77, 0.41)	−5.18 (− 8.34, − 2.02)	− 3.94 (− 7.07, − 0.82)		−5.03 (−7.15, − 2.91)	− 4.57 (− 6.75, − 2.39)	
HDL-cholesterol, mg/dl					0.82			0.30
Women	2.61 (1.67, 3.55)	2.02 (1.30, 2.75)	2.55 (1.64, 3.46)	1.82 (0.94, 2.71)		2.25 (1.68, 2.82)	2.18 (1.44, 2.92)	
Men	0.65 (− 0.33, 1.64)	0.58 (− 0.42, 1.58)	1.48 (0.38, 2.59)	0.49 (− 0.46, 1.43)		1.21 (0.47, 1.94)	0.45 (− 0.34, 1.25)	
Triglycerides, mg/dl^c^					0.09			0.09
Women	0.92 (0.88, 0.96)	0.91 (0.87, 0.95)	0.96 (0.92, 1.00)	0.95 (0.91, 0.99)		0.94 (0.91, 0.96)	0.93 (0.89, 0.97)	
Men	0.98 (0.93, 1.03)	0.95 (0.91, 1.00)	0.92 (0.87, 0.97)	0.95 (0.91, 1.00)		0.93 (0.89, 0.96)	0.97 (0.94, 1.01)	
Fasting glucose, mg/dl					0.62			0.23
Women	3.43 (2.04, 4.83)	3.55 (2.46, 4.64)	4.18 (2.78, 5.59)	4.90 (3.66, 6.14)		4.56 (3.68, 5.43)	3.06 (2.12, 4.00)	
Men	3.87 (2.54, 5.20)	4.70 (3.10, 6.31)	4.40 (2.96, 5.85)	4.22 (2.48, 5.97)		4.52 (3.37, 5.68)	4.11 (3.08, 5.14)	
2-h glucose, mg/dl					0.31			0.56
Women	8.13 (4.59, 11.68)	9.00 (6.03, 11.97)	8.38 (5.11, 11.65)	13.24 (9.82, 16.66)		10.08 (8.02, 12.15)	8.91 (6.26, 11.57)	
Men	5.95 (2.61, 9.29)	7.40 (3.20, 11.59)	6.78 (1.31, 12.24)	6.13 (2.43, 9.83)		6.48 (3.54, 9.41)	6.65 (3.85, 9.46)	
HbA1c, mg/dl					0.48			0.45
Women	0.19 (0.15, 0.23)	0.18 (0.15, 0.21)	0.19 (0.15, 0.22)	0.17 (0.14, 0.20)		0.18 (0.16, 0.20)	0.19 (0.15, 0.22)	
Men	0.18 (0.14, 0.22)	0.19 (0.14, 0.23)	0.13 (0.09, 0.17)	0.15 (0.11, 0.20)		0.15 (0.12, 0.18)	0.18 (0.14, 0.21)	
Fasting insulin, mU/L^c^					0.14			**0.01**
Women	1.16 (1.10, 1.22)	1.20 (1.14, 1.26)	1.23 (1.18, 1.29)	1.25 (1.20, 1.31)		**1.25 (1.21, 1.29)**	**1.15 (1.11, 1.20)**	
Men	1.19 (1.13, 1.26)	1.26 (1.17, 1.35)	1.16 (1.07, 1.25)	1.20 (1.13, 1.27)		**1.19 (1.14, 1.24)**	**1.21 (1.15, 1.27)**	
HOMA-IR^c^					0.21			**0.01**
Women	1.21 (1.14, 1.28)	1.23 (1.16, 1.30)	1.27 (1.22, 1.33)	1.30 (1.23, 1.36)		**1.29 (1.24, 1.33)**	**1.20 (1.14, 1.25)**	
Men	1.25 (1.18, 1.33)	1.32 (1.22, 1.43)	1.22 (1.13, 1.33)	1.27 (1.19, 1.35)		**1.25 (1.20, 1.31)**	**1.27 (1.21, 1.34)**	

^a^All models adjusted for sex, use of medications that affect the dependent variable at baseline and/or visit2, baseline levels of the dependent variable, and elapsed time between visits, household income, education, employment status, Hispanic/Latino background, field center, and nativity status, smoking, alcohol consumption, health insurance status, healthcare utilization, self-reported health, diet quality (all assessed at baseline), change in health insurance coverage, BMI and waist-to-hip ratio (except for change in BMI and waist circumference as outcomes). ^b^Meeting 2018 physical activity guidelines defined using measured activity scaled to 7 days of accelerometer wear as 150 minutes/week moderate intensity physical activity, 75 minutes/week vigorous intensity activity, or an equivalent combination of both. ^c^ Geometric means (95% CI) presented for triglycerides, fasting insulin, HOMA-IR; significant findings <0.05 bolded

We further examined the association of meeting MVPA guidelines with changes in fasting insulin and HOMA-IR by Hispanic/Latino background (Table 6.) We found significant interaction with changes in fasting insulin and marginal interaction with changes in HOMA-IR by Hispanic/Latino background. Background stratified analyses showed Dominicans who met MVPA guidelines, had significantly lower increases in these biomarkers over the six-year period. South Americans also indicated a trend for lower increase in these biomarkers; other groups, except Mexicans, also reflected lower increase in these biomarkers; however, the results were not statistically significant and the confidence intervals were somewhat wide.

**Table 6:** Multivariable-adjusted mean changes in fasting insulin & HOMA-IR (95% CI) over 6 years of follow-up, according to meeting guidelines for moderate-to-vigorous physical activity by Hispanic/Latino background – n ~ 7900^a^

Change in CVD risk factors	MVPA guidelines^b^		P	P of inter-action
	Not meet	Meet		
log Fasting insulin, mg/dl				**0.01**
Dominican	**0.25 (0.15, 0.35)**	**0.07 (−0.03, 0.17)**	**0.006**	
Central American	0.21 (0.15, 0.27)	0.12 (0.02, 0.22)	0.59	
Cuban	0.14 (0.06, 0.22)	0.06 (− 0.06, 0.18)	0.14	
Mexican	0.23 (0.17, 0.29)	0.24 (0.18, 0.30)	0.68	
Puerto Rican	0.19 (0.11, 0.27)	0.13 (0.05, 0.21)	0.25	
South American	0.18 (0.10, 0.26)	0.10 (0.00, 0.20)	0.05	
Mixed/other	0.25 (0.11, 0.39)	0.44 (0.24, 0.64)	0.09	
log HOMA-IR				**0.04**
Dominican	**0.29 (0.17, 0.41)**	**0.12 (0.02, 0.22)**	**0.03**	
Central American	0.24 (0.16, 0.32)	0.15 (0.03, 0.27)	0.73	
Cuban	0.15 (0.07, 0.23)	0.07 (−0.05, 0.19)	0.13	
Mexican	0.28 (0.22, 0.34)	0.29 (0.23, 0.35)	0.89	
Puerto Rican	0.26 (0.16, 0.36)	0.18 (0.08, 0.28)	0.25	
South American	0.21 (0.13, 0.29)	0.12 (0.00, 0.24)	0.08	
Mixed/other	**0.29 (0.13, 0.45)**	**0.52 (0.30, 0.74)**	**0.02**	

^a^All models adjusted for sex, use of medications that affect the dependent variable at baseline and/or visit2, baseline levels of the dependent variable, and elapsed time between visits, household income, education, employment status, Hispanic/Latino background, field center, and nativity status, smoking, alcohol consumption, health insurance status, healthcare utilization, self-reported health, diet quality (all assessed at baseline), change in health insurance coverage, BMI and waist-to-hip ratio. Significant findings *p* < 0.05 bolded

^b^Meeting 2018 physical activity guidelines defined using measured activity scaled to 7 days of accelerometer wear as 150 minutes/week moderate intensity physical activity, 75 minutes/week vigorous intensity activity, or an equivalent combination of both

No significant findings were apparent when we assessed multivariable-adjusted mean changes in cardiovascular disease risk factors (95% CI) over six years of follow-up, according to joint distribution of quartiles of sedentary behavior and meeting guidelines for MVPA (Additional Table 4). We did not find heterogeneity effect of MVPA in associations with change in cardiovascular biomarkers across sedentary quartiles.

## Discussion

In summary we find that for all individuals whether with normal glycemic levels, or prediabetes, meeting MVPA guidelines at V1 was associated with significantly lower rise in level of fasting glucose in adjusted models. Focusing only on the healthiest group or adults with normal glycemic levels without cardiovascular disease, we find that the least sedentary adults had a higher level of decrease in mean LDL-cholesterol and lower mean increase in HbA1c over the six -year period compared to adults who were most sedentary. This study is novel in reporting sedentary behavior, MVPA and changes in cardiometabolic risk in Hispanic/Latinos, evidence which is virtually non-existent. In this study adults with lesser volume of MVPA had increases in all the cardiometabolic biomarkers over time except for LDL cholesterol; these increases were not statistically significantly different from adults meeting MVPA guidelines except for fasting glucose in the overall group and in fasting glucose and HOMA-IR in the healthiest group (normoglycemic without cardiovascular disease.) These data suggest that an active lifestyle may blunt the association of advancing age with worsening cardiometabolic risk factors [34].

The finding of overall reduction in LDL cholesterol from V1 to V2 is interesting, given the fact that we adjusted our models for lipid lowering medications and may be related to the beginning of the elimination of trans fats from the food supply in the US initiated by Food and Drug Administration (FDA) in 2015 which is during the time period between V1 to V2. FDA provided three years for the food manufacturers and later an extension to January 1, 2020 to come up with alternatives to trans fats [35]. This action was prompted by research associating trans fats intake to increase in harmful levels of LDL-cholesterol and cardiovascular disease [36].

While exploratory subgroup analyses were made possible by the large size of our cohort, the results may possibly be due to chance. In contrast with results associated with MVPA, lower levels of sedentary behavior were not associated with changes in cardiometabolic biomarkers. However, among the group without cardiovascular disease, prediabetes or diabetes at V1, lower levels of sedentary behavior were associated with higher decrease (or lower increase) over time in LDL-cholesterol and lower increases in HbA1c levels than individuals who were more sedentary. Therefore reduction in sedentary behavior may be especially important from a primary prevention perspective. On the other hand, the level of physical activity needed, whether lower sedentary behavior or higher MVPA to achieve change, was not detected or were too low in the least healthy groups. In addition, although we adjusted for use of medications to treat dyslipidemia and hypertension at both visits, it is possible that the use of pharmacological and non-pharmacological interventions (e.g., weight loss and diet modification) in the population affected by cardiometabolic conditions may have obscured, confounded, or eliminated the association between MVPA and metabolic biomarkers.

Another interesting finding that has clinical applications was that on average women, but not men meeting MVPA guidelines, experienced reductions over time in both biomarkers fasting insulin and HOMA-IR. The lack of association between meeting MVPA guidelines and these cardiometabolic biomarkers in men merits additional study. Focusing on the group restricted to adults with prediabetes, it is not clear why they do not seem to reap any benefits from meeting MVPA or having lower levels of sedentary behavior. It is possible that individuals with prediabetes who maintained a high level of MVPA at V1 and at V2, may have slowed their progression to diabetes [37].

Limitations of the study include that the Hispanics/Latinos in this study are primarily from urban areas and may not represent Hispanics/Latinos who reside in rural areas and who may have different patterns of physical activity.

To reduce selection bias with respect to the approximately 20% participants did not adhere to the Actical protocol, we adjusted the sampling weights using inverse probability weights to account for observed differences between adherent and non-adherent participants. We were also limited to a single measure of accelerometry. Another limitation is from the accelerometry device itself that may not capture all types of activity such as weight lifting [38]. We also assessed physical activity over a week to limit participant burden. An additional study limitation is that while we had a diverse cohort, sample sizes for some subgroups were not large. The finding that the Dominican group derived particular MVPA related benefits by lower increases in levels of fasting insulin and HOMA-IR and that they might be different from other Hispanics/Latinos in this regard, needs replication. With respect to the magnitude of the effects observed, while relatively small (e.g. mean increase of 3.46 mg/dl of fasting glucose for all adults meeting MVPA guidelines) over the life course the cumulative effects of small changes in lifestyle could have substantial downstream effects on risks for cardiometabolic diseases.

## Conclusion

In conclusion, this study highlights the importance of MVPA in a diverse group of Hispanics/Latinos. Findings were especially strong among women, and although we were unable to explain this finding, it raises hypotheses about biological differences in metabolism, and response to exercise, or lifestyle patterns between men and women. The most marked benefits from an active lifestyle were found in relation to fasting insulin and HOMA-IR and highlight the potential of behavioral intervention to prevent development and worsening of insulin resistance. Low levels of sedentary behavior, on the other hand, were associated with health benefits especially for individuals free of cardiometabolic disease.

## Supplementary information

**Additional file 1: Table S1.** Characteristics of participants by Actical adherence (among all participants who attended V2).

**Additional file 2: Table S2.** Multivariable-adjusted mean changes in cardiovascular disease risk factors (95% CI) over 6 years of follow-up among individuals with prediabetes at baseline, according to quartiles of sedentary time and meeting guidelines for moderate-to-vigorous physical activity (N ~ 3301, 2 h glucose N ~ 2733).

**Additional file 3: Table S3.** LDL Cholesterol & Statin use.

**Additional file 4: Table S4.** Multivariable-adjusted mean changes in cardiovascular disease risk factors (95% CI) over 6 years of follow-up, according to cross-classification of quartiles of sedentary time and meeting guidelines for moderate-to-vigorous physical activity.

## Data Availability

The Biologic Specimen and Data Repository Information Coordinating Center (BioLINCC) serves as the data repository. center for HCHS/SOL: //biolincc.nhlbi.nih.gov/home. Accession number: HLB01141418a.

## Abbreviations

BMI: Body Mass Index
FDA: Food and Drug Administration
HBA1c: Hemoglobin A1c
HDL: High density lipoprotein
HOMA-IR: Homeostatic model assessment of insulin resistance
LDL: Low density lipoproteins
MVPA: Moderate-to-Vigorous Physical Activity
PA: Physical activity
V1: Visit 1
V2: Visit 2

## References

[1] Matthews CE, Chen KY, Freedson PS, Buchowski MS, Beech BM, Pate RR, Troiano RP (2008). Amount of time spent in sedentary behaviors in the United States, 2003-2004. Amer J Epidemiol.

[2] Owen N, Healy GN, Matthews CE, Dunstan DW (2010). Too much sitting: the population health science of sedentary behavior. Exercise Sports Sci Rev.

[3] Hispanic Heritage Month 2019 [https://www.census.gov/newsroom/facts-for-features/2019/hispanic-heritage-month.html]. Accessed 6 Aug 2020.

[4] Schneiderman N, Llabre M, Cowie CC, Barnhart J, Carnethon M, Gallo LC, Giachello AL, Heiss G, Kaplan RC, LaVange LM (2014). Prevalence of diabetes among Hispanics/Latinos from diverse backgrounds: the Hispanic community health study/study of Latinos (HCHS/SOL). Diabetes Care.

[5] Marquez DX, Neighbors CJ, Bustamante EE (2010). Leisure time and occupational physical activity among racial or ethnic minorities. Med Sci Sports Exer.

[6] Echeverría SE, Divney A, Rodriguez F, Sterling M, Vasquez E, Murillo R, Lopez L (2019). Nativity and occupational determinants of physical activity participation among Latinos. Amer J Prev Med.

[7] Physical Activity Guidelines for Americans [https://health.gov/paguidelines/second-edition/pdf/Physical_Activity_Guidelines_2nd_edition.pdf ]. Accessed 9 May 2019.

[8] Tremblay MS, Aubert S, Barnes JD, Saunders TJ, Carson V, Latimer-Cheung AE, Chastin SFM, Altenburg TM, Chinapaw MJM (2017). Sedentary behavior research network (SBRN) - terminology consensus project process and outcome. Int J Behav Nutr Phys Act.

[9] Young DR, Hivert MF, Alhassan S, Camhi SM, Ferguson JF, Katzmarzyk PT, Lewis CE, Owen N, Perry CK, Siddique J (2016). Sedentary behavior and cardiovascular morbidity and mortality: a science advisory from the American Heart Association. Circulation.

[10] Ohlsson C, Gidestrand E, Bellman J, Larsson C, Palsdottir V, Hägg D, Jansson PA, Jansson JO (2020). Increased weight loading reduces body weight and body fat in obese subjects - a proof of concept randomized clinical trial. EClinicalMedicine.

[11] Tremblay MS, Colley RC, Saunders TJ, Healy GN, Owen N (2010). Physiological and health implications of a sedentary lifestyle. Appl Physiol Nutr Metab.

[12] Henson J, Yates T, Biddle SJ, Edwardson CL, Khunti K, Wilmot EG, Gray LJ, Gorely T, Nimmo MA, Davies MJ (2013). Associations of objectively measured sedentary behaviour and physical activity with markers of cardiometabolic health. Diabetologia.

[13] Sisson SB, Camhi SM, Church TS, Martin CK, Tudor-Locke C, Bouchard C, Earnest CP, Smith SR, Newton RL, Rankinen T (2009). Leisure time sedentary behavior, occupational/domestic physical activity, and metabolic syndrome in U.S. men and women. Metab Syndr Relat Disord.

[14] Fuzeki E, Engeroff T, Banzer W (2017). Health Benefits of Light-Intensity Physical Activity: A Systematic Review of Accelerometer Data of the National Health and Nutrition Examination Survey (NHANES). Sports Med (Auckland, NZ).

[15] Australia's Physical Activity and Sedentary Behaviour Guidelines and the Australian 24-Hour Movement Guidelines [https://www1.health.gov.au/internet/main/publishing.nsf/Content/health-pubhlth-strateg-phys-act-guidelines]. Accessed 22 Nov 2019.

[16] Merchant G, Buelna C, Castaneda SF, Arredondo EM, Marshall SJ, Strizich G, Sotres-Alvarez D, Chambers EC, McMurray RG, Evenson KR (2015). Accelerometer-measured sedentary time among Hispanic adults: results from the Hispanic community health study/study of Latinos (HCHS/SOL). Prev Med Rep.

[17] Qi Q, Strizich G, Merchant G, Sotres-Alvarez D, Buelna C, Castaneda SF, Gallo LC, Cai J, Gellman MD, Isasi CR (2015). Objectively measured sedentary time and Cardiometabolic biomarkers in US Hispanic/Latino adults: the Hispanic community health study/study of Latinos (HCHS/SOL). Circulation.

[18] Ormazabal V, Nair S, Elfeky O, Aguayo C, Salomon C, Zuñiga FA (2018). Association between insulin resistance and the development of cardiovascular disease. Cardiovasc Diabetol.

[19] Hands B, Parker H, Larkin D, Cantell M, Rose E (2016). Male and Female Differences in Health Benefits Derived from Physical Activity: Implications for Exercise Prescription. J Womens Health, Issues Care.

[20] Lavange LM, Kalsbeek WD, Sorlie PD, Aviles-Santa LM, Kaplan RC, Barnhart J, Liu K, Giachello A, Lee DJ, Ryan J (2010). Sample design and cohort selection in the Hispanic community health study/study of Latinos. Ann Epidemiol.

[21] Sorlie PD, Aviles-Santa LM, Wassertheil-Smoller S, Kaplan RC, Daviglus ML, Giachello AL, Schneiderman N, Raij L, Talavera G, Allison M (2010). Design and implementation of the Hispanic community health study/study of Latinos. Ann Epidemiol.

[22] Motel S, Patten E. Pew research center: characteristics of the 60 largest metropolitan areas by Hispanic concentration [https://www.pewhispanic.org/2012/09/19/appendix-a-3/]. Accessed 9 May 2019.

[23] Evenson KR, Sotres-Alvarez D, Deng YU, Marshall SJ, Isasi CR, Esliger DW, Davis S (2015). Accelerometer adherence and performance in a cohort study of US Hispanic adults. Med Sci Sports Exerc.

[24] Arredondo EM, Sotres-Alvarez D, Stoutenberg M, Davis SM, Crespo NC, Carnethon MR, Castaneda SF, Isasi CR, Espinoza RA, Daviglus ML (2016). Physical activity levels in U.S. Latino/Hispanic adults: results from the Hispanic community health study/study of Latinos. Am J Prev Med.

[25] Choi L, Liu Z, Matthews CE, Buchowski MS (2011). Validation of accelerometer wear and nonwear time classification algorithm. Med Sci Sports Exerc.

[26] Colley RC, Garriguet D, Janssen I, Craig CL, Clarke J, Tremblay MS (2011). Physical activity of Canadian adults: accelerometer results from the 2007 to 2009 Canadian health measures survey. Health Rep.

[27] Colley RC, Tremblay MS (2011). Moderate and vigorous physical activity intensity cut-points for the Actical accelerometer. J Sports Sci.

[28] Wong SL, Colley R, Connor Gorber S, Tremblay M (2011). Actical accelerometer sedentary activity thresholds for adults. J Phys Act Health.

[29] Thyagarajan B, Howard AG, Durazo-Arvizu R, Eckfeldt JH, Gellman MD, Kim RS, Liu K, Mendez AJ, Penedo FJ, Talavera GA (2016). Analytical and biological variability in biomarker measurement in the Hispanic community health study/study of Latinos. Clin Chim Acta.

[30] Daviglus ML, Talavera GA, Aviles-Santa ML, Allison M, Cai J, Criqui MH, Gellman M, Giachello AL, Gouskova N, Kaplan RC, et al. Prevalence of major cardiovascular risk factors and cardiovascular diseases among Hispanic/Latino individuals of diverse backgrounds in the United States. JAMA. 2012;308(17):1775–84. 10.1001/jama.2012.14517 PMC3777250 23117778

[31] Yokoyama H, Emoto M, Fujiwara S, Motoyama K, Morioka T, Komatsu M, Tahara H, Shoji T, Okuno Y, Nishizawa Y (2003). Quantitative insulin sensitivity check index and the reciprocal index of homeostasis model assessment in normal range weight and moderately obese type 2 diabetic patients. Diabetes Care.

[32] Healy GN, Matthews CE, Dunstan DW, Winkler EA, Owen N (2011). Sedentary time and cardio-metabolic biomarkers in US adults: NHANES 2003-06. Eur Heart J.

[33] Vasquez E, Strizich G, Gallo L, Marshall SJ, Merchant GC, Murillo R, Penedo FJ, Salazar C, Sotres-Alvarez D, Shaw BA (2016). The role of stress in understanding differences in sedentary behavior in Hispanic/Latino adults: results from the Hispanic community health study/study of Latinos sociocultural ancillary study. J Phys Act Health.

[34] Kaplan RC, Aviles-Santa ML, Parrinello CM, Hanna DB, Jung M, Castaneda SF, Hankinson AL, Isasi CR, Birnbaum-Weitzman O, Kim RS, et al. Body mass index, sex, and cardiovascular disease risk factors among Hispanic/Latino adults: Hispanic community health study/study of Latinos. J Am Heart Assoc. 2014;3(4):e000923. 10.1161/JAHA.114.000923 PMC5680614 25008353

[35] Trans Fat- FDA [https://www.fda.gov/food/food-additives-petitions/trans-fat]. Accessed 2 Mar 2020.

[36] Mozaffarian D, Katan MB, Ascherio A, Stampfer MJ, Willett WC (2006). Trans fatty acids and cardiovascular disease. New Engl J Med.

[37] Glechner A, Keuchel L, Affengruber L, Titscher V, Sommer I, Matyas N, Wagner G, Kien C, Klerings I, Gartlehner G (2018). Effects of lifestyle changes on adults with prediabetes: a systematic review and meta-analysis. Prim Care Diabetes.

[38] Shaw PA, McMurray R, Butte N, Sotres-Alvarez D, Sun H, Stoutenberg M, Evenson KR, Wong WW, Moncrieft AE, Sanchez-Johnsen LAP (2019). Calibration of activity-related energy expenditure in the Hispanic community health study/study of Latinos (HCHS/SOL). J Sci Med Sport.

