# Supplementary material for: Are sedentary behavior and physical activity independently associated with cardiometabolic benefits? The Hispanic Community Health Study/Study of Latinos
Source: BMC Public Health. 2020 Sep 14;20:1400. doi: 10.1186/s12889-020-09497-5 (PMC7490882; doi:10.1186/s12889-020-09497-5)
Supplement: Supplementary file 1 — Additional file 1: Table S1. Characteristics of participants by Actical adherence (among all participants who attended V2). [file 12889_2020_9497_MOESM1_ESM.docx]

**Additional Table 1**: Characteristics of participants by Actical adherence (among all participants who attended V2)

|  | **Non-adherent** | **Adherent (≥10hrs, ≥3 days)** | **p-value** |
| --- | --- | --- | --- |
| No. of participants | 2283 | 9340 |  |
| Sex, % male | 45.9 ( 43.2, 48.6) | 48.5 ( 47.0, 49.9) | 0.1 |
| **Age in years, %** |  |  | <0.0001 |
| 18-44 | 68.1 ( 65.3, 70.8) | 57.3 ( 55.5, 59.1) |  |
| 45-64 | 26.1 ( 24.0, 28.4) | 33.4 ( 31.9, 34.8) |  |
| 65-76 | 5.8 ( 4.5, 7.5) | 9.3 ( 8.4, 10.4) |  |
| **Hispanic background, %** |  |  | <0.0001 |
| Dominican | 8.7 ( 7.0, 10.8) | 10.3 ( 8.9, 11.9) |  |
| Central American | 8.3 ( 6.6, 10.4) | 7.4 ( 6.2, 8.7) |  |
| Cuban | 23.9 ( 20.0, 28.3) | 18.9 ( 16.1, 22.1) |  |
| Mexican | 32.3 ( 28.3, 36.4) | 38.8 ( 35.6, 42.0) |  |
| Puerto Rican | 15.5 ( 13.1, 18.3) | 16.1 ( 14.5, 17.8) |  |
| South American | 5.3 ( 4.0, 6.9) | 4.7 ( 4.1, 5.4) |  |
| Other/>1 | 6.0 ( 4.5, 7.9) | 3.9 ( 3.2, 4.7) |  |
| Born in 50 states, % | 28.0 ( 24.8, 31.4) | 21.0 ( 19.3, 22.7) | <0.0001 |
| ≥ High school education, % | 70.8 ( 67.9, 73.6) | 66.9 ( 65.2, 68.6) | 0.015 |
| **Annual family income, %** |  |  | 0.59 |
| <20,000$ | 48.3 ( 44.7, 51.9) | 46.9 ( 44.7, 49.0) |  |
| 20,000-50,000$ | 38.9 ( 35.6, 42.4) | 40.8 ( 39.0, 42.7) |  |
| >50,000$ | 12.8 ( 10.4, 15.6) | 12.3 ( 10.8, 13.9) |  |
| Employed full/part-time, % | 47.1 ( 44.0, 50.2) | 52.7 ( 51.0, 54.4) | 0.001 |
| Health insurance, % | 50.1 ( 47.1, 53.2) | 51.2 ( 49.1, 53.3) | 0.5 |
| Currently smokes, % | 22.4 ( 20.0, 25.0) | 19.1 ( 17.7, 20.6) | 0.019 |
| **Alcohol use level, %** |  |  | 0.2 |
| Non-drinker | 48.0 ( 44.7, 51.3) | 49.3 ( 47.5, 51.1) |  |
| Moderate drinker | 44.9 ( 41.6, 48.3) | 45.2 ( 43.4, 47.1) |  |
| Problem drinker* | 7.1 ( 5.5, 9.0) | 5.4 ( 4.7, 6.3) |  |
| Antidiabetic medication, % | 7.5 ( 6.2, 9.0) | 8.8 ( 8.1, 9.6) | 0.11 |
| Antihypertensive medication, % | 15.6 ( 13.6, 17.9) | 17.3 ( 16.1, 18.5) | 0.18 |
| Lipid-lowering drugs, % | 8.5 ( 7.3, 10.0) | 9.9 ( 9.1, 10.7) | 0.11 |
| Alternative healthy eating index, mean | 46.2 ( 45.8, 46.7) | 48.0 ( 47.7, 48.4) | <0.0001 |
| Physical health score (SF-12), mean | 49.7 ( 49.1, 50.3) | 49.8 ( 49.5, 50.2) | 0.69 |
| Total self-reported physical activity, min | 133.8 (121.5, 146.1) | 138.1 (130.9, 145.4) | 0.54 |
| Hypertension | 22.5 ( 20.1, 25.0) | 24.7 ( 23.3, 26.1) | 0.1 |
| Prediabetes | 31.9 ( 29.4, 34.6) | 36.3 ( 34.9, 37.7) | 0.028 |
| Diabetes | 14.8 ( 12.9, 16.9) | 15.1 ( 14.2, 16.2) | 0.24 |
| Dyslipidemia | 39.4 ( 36.5, 42.3) | 38.5 ( 37.0, 40.0) | 0.59 |
| Change in SBP (v2 - v1), mmHg | 1.1 ( 0.4, 1.9) | 1.2 ( 0.7, 1.6) | 0.95 |
| Change in DBP (v2 - v1), mmHg | -0.09 ( -0.7, 0.5) | -0.03 ( -0.4, 0.3) | 0.88 |
| Change in total cholesterol, mg/dL | -4.0 ( -6.3, -1.7) | -5.3 ( -6.5, -4.2) | 0.32 |
| Change in triglycerides (v2 - v1), mg/dL | 0.9 ( 0.9, 1.0) | 0.9 ( 0.9, 1.0) | 0.87 |
| Change in HDL (v2 - v1), mg/dL | 1.4 ( 0.8, 2.1) | 1.6 ( 1.3, 2.0) | 0.62 |
| Change in LDL (v2 - v1), mg/dL | -4.4 ( -6.3, -2.4) | -5.6 ( -6.6, -4.6) | 0.26 |
| Change in fasting glucose (v2 - v1), mg/dL | 5.7 ( 3.6, 7.8) | 4.9 ( 4.0, 5.8) | 0.52 |
| Change in 2-h glucose (v2 - v1), mg/dL | 8.3 ( 5.3, 11.3) | 9.4 ( 8.0, 10.8) | 0.53 |
| Change in HbA1c (v2 - v1), % | 0.22 ( 0.17, 0.28) | 0.20 ( 0.17, 0.22) | 0.4 |
| Change in fasting insulin (v2 - v1), mU/L | 1.17 ( 1.12, 1.22) | 1.20 ( 1.18, 1.23) | 0.26 |
| Change in HOMA_ir (v2 - v1) | 1.22 ( 1.17, 1.28) | 1.25 ( 1.22, 1.28) | 0.38 |
| V1 SBP, mmHg | 118.2 (117.1, 119.2) | 120.1 (119.5, 120.6) | 0.0008 |
| V1 DBP, mmHg | 72.3 ( 71.6, 73.1) | 72.0 ( 71.7, 72.4) | 0.36 |
| V1 total cholesterol, mg/dL | 192.4 (189.7, 195.1) | 195.3 (193.9, 196.8) | 0.06 |
| V1 triglycerides, mg/dL | 109.8 (106.2, 113.4) | 111.8 (109.7, 113.9) | 0.30 |
| V1 HDL, mg/dL | 48.2 ( 47.5, 49.0) | 48.6 ( 48.1, 49.0) | 0.43 |
| V1 LDL, mg/dL | 118.5 (116.1, 120.9) | 120.7 (119.5, 121.9) | 0.12 |
| V1 fasting glucose, mg/dL | 102.2 (100.1, 104.3) | 102.3 (101.2, 103.3) | 0.93 |
| V1 2-h glucose, mg/dL | 115.9 (113.2, 118.6) | 117.7 (116.4, 119.0) | 0.24 |
| V1 HbA1c, % | 5.74 ( 5.67, 5.81) | 5.75 ( 5.71, 5.78) | 0.92 |
| V1 fasting insulin, mU/L | 10.68 (10.12, 11.26) | 10.24 ( 9.99, 10.50) | 0.13 |
| V1 HOMA-IR | 2.60 ( 2.45, 2.75) | 2.51 ( 2.44, 2.58) | 0.22 |
| V2 SBP, mmHg | 119.3 (118.3, 120.3) | 121.2 (120.6, 121.8) | 0.0004 |
| V2 DBP, mmHg | 72.2 ( 71.6, 72.9) | 72.0 ( 71.6, 72.3) | 0.46 |
| V2 total cholesterol, mg/dL | 188.5 (186.0, 191.1) | 190.1 (188.8, 191.4) | 0.28 |
| V2 triglycerides, mg/dL | 103.7 (99.95, 107.7) | 105.6 (103.5, 107.8) | 0.38 |
| V2 HDL, mg/dL | 49.6 ( 48.7, 50.6) | 50.2 ( 49.7, 50.7) | 0.33 |
| V2 LDL, mg/dL | 114.5 (112.1, 116.8) | 114.9 (113.8, 116.1) | 0.72 |
| V2 fasting glucose, mg/dL | 107.6 (105.2, 109.9) | 107.2 (106.2, 108.1) | 0.74 |
| V2 2-h glucose, mg/dL | 119.2 (116.2, 122.2) | 123.8 (122.4, 125.1) | 0.01 |
| V2 HbA1c, % | 5.97 ( 5.89, 6.05) | 5.94 ( 5.91, 5.98) | 0.60 |
| V2 fasting insulin, mU/L | 12.35 (11.76, 12.97) | 12.28 (12.01, 12.55) | 0.83 |
| V2 HOMA-IR | 3.14 ( 2.97, 3.32) | 3.13 ( 3.05, 3.21) | 0.91 |
