# Supplementary material for: Are sedentary behavior and physical activity independently associated with cardiometabolic benefits? The Hispanic Community Health Study/Study of Latinos
Source: BMC Public Health. 2020 Sep 14;20:1400. doi: 10.1186/s12889-020-09497-5 (PMC7490882; doi:10.1186/s12889-020-09497-5)
Supplement: Supplementary file 2 — Additional file 2: Table S2. Multivariable-adjusted mean changes in cardiovascular disease risk factors (95% CI) over 6 years of follow-up among individuals with prediabetes at baseline, according to quartiles of sedentary time and meeting guidelines for moderate-to-vigorous physical activity (N ~ 3301, 2 h glucose N ~ 2733). [file 12889_2020_9497_MOESM2_ESM.docx]

**Additional Table 2**: Multivariable-adjusted mean changes in cardiovascular disease risk factors (95% CI) over 6 years of follow-up among individuals with prediabetes at baseline, according to quartiles of sedentary time and meeting guidelines for moderate-to-vigorous physical activity (N~3301, 2h glucose N~2733)

|  | **Sedentary time** | | | |  | **MVPA guidelines*** | |  |
| --- | --- | --- | --- | --- | --- | --- | --- | --- |
| **Change in CVD risk factors** | **Quartile 1**  (0.8-10.8 h) | **Quartile 2**  (10.8-11.9 h) | **Quartile 3**  (11.9-12.9 h) | **Quartile 4**  (12.9-16.0 h) | ***P-trend*** | **Not meet** | **Meet** | ***P*** |
| BMI, kg/m2 |  |  |  |  |  |  |  |  |
| Model 1 | 0.60 ( 0.31, 0.89) | 0.79 ( 0.42, 1.16) | 0.24 ( 0.01, 0.48) | 0.37 ( 0.14, 0.60) | 0.07 | 0.50 ( 0.33, 0.66) | 0.50 ( 0.26, 0.75) | 0.96 |
| Model 2 | 0.54 ( 0.26, 0.83) | 0.77 ( 0.43, 1.11) | 0.24 ( 0.00, 0.49) | 0.43 ( 0.20, 0.65) | 0.21 | 0.48 ( 0.30, 0.65) | 0.52 ( 0.29, 0.74) | 0.77 |
| Model 3 | 0.50 ( 0.21, 0.79) | 0.77 ( 0.43, 1.11) | 0.26 ( 0.01, 0.50) | 0.46 ( 0.23, 0.69) | 0.42 | 0.50 ( 0.31, 0.69) | 0.49 ( 0.26, 0.72) | 0.94 |
| Waist circumference, cm |  |  |  |  |  |  |  |  |
| Model 1 | 2.42 ( 1.68, 3.16) | 2.10 ( 1.03, 3.16) | 1.60 ( 0.80, 2.40) | 2.49 ( 1.63, 3.36) | 0.79 | 2.21 ( 1.69, 2.72) | 2.07 ( 1.35, 2.79) | 0.73 |
| Model 2 | 2.55 ( 1.79, 3.30) | 2.20 ( 1.21, 3.20) | 1.60 ( 0.77, 2.43) | 2.28 ( 1.45, 3.12) | 0.41 | 2.42 ( 1.86, 2.98) | 1.81 ( 1.11, 2.50) | 0.13 |
| Model 3 | 2.72 ( 1.93, 3.52) | 2.21 ( 1.22, 3.20) | 1.54 ( 0.70, 2.38) | 2.15 ( 1.29, 3.01) | 0.22 | 2.47 ( 1.88, 3.06) | 1.74 ( 0.99, 2.48) | 0.12 |
| Systolic BP, mmHg |  |  |  |  |  |  |  |  |
| Model 1 | -0.18 (-1.29, 0.93) | 1.54 ( 0.24, 2.84) | 0.05 (-1.37, 1.48) | 0.28 (-0.97, 1.52) | 0.85 | 0.37 (-0.60, 1.33) | 0.51 (-0.44, 1.45) | 0.83 |
| Model 2 | -0.18 (-1.37, 1.01) | 1.51 ( 0.23, 2.78) | 0.17 (-1.21, 1.54) | 0.08 (-1.15, 1.31) | 0.96 | 0.50 (-0.44, 1.44) | 0.24 (-0.74, 1.22) | 0.68 |
| Model 3 | -0.12 (-1.40, 1.17) | 1.53 ( 0.24, 2.82) | 0.19 (-1.18, 1.56) | -0.03 (-1.28, 1.22) | 0.86 | 0.46 (-0.51, 1.42) | 0.30 (-0.72, 1.31) | 0.81 |
| Diastolic BP, mmHg |  |  |  |  |  |  |  |  |
| Model 1 | -1.73 (-2.53, -0.94) | -0.50 (-1.46, 0.45) | -1.34 (-2.25, -0.44) | -0.68 (-1.62, 0.26) | 0.16 | -1.28 (-1.94, -0.61) | -0.78 (-1.50, -0.05) | 0.30 |
| Model 2 | -1.68 (-2.48, -0.88) | -0.42 (-1.37, 0.52) | -1.23 (-2.12, -0.33) | -1.04 (-1.97, -0.12) | 0.41 | -1.01 (-1.69, -0.33) | -1.21 (-1.92, -0.51) | 0.66 |
| Model 3 | -1.62 (-2.48, -0.77) | -0.42 (-1.37, 0.53) | -1.25 (-2.14, -0.36) | -1.08 (-2.00, -0.16) | 0.56 | -1.07 (-1.75, -0.39) | -1.13 (-1.88, -0.38) | 0.91 |
| LDL-cholesterol, mg/dl |  |  |  |  |  |  |  |  |
| Model 1 | -5.12 (-7.81, -2.43) | -5.82 (-9.99, -1.64) | -7.65 (-10.8, -4.49) | -8.28 (-10.8, -5.81) | 0.07 | -6.86 (-8.77, -4.95) | -6.51 (-9.23, -3.78) | 0.81 |
| Model 2 | -5.80 (-8.59, -3.01) | -6.13 (-9.85, -2.40) | -7.25 (-10.3, -4.20) | -7.76 (-10.3, -5.22) | 0.30 | -6.37 (-8.43, -4.32) | -7.21 (-9.79, -4.63) | 0.55 |
| Model 3 | -6.58 (-9.47, -3.69) | -6.07 (-9.81, -2.34) | -6.82 (-9.93, -3.72) | -7.46 (-10.0, -4.88) | 0.66 | -6.17 (-8.33, -4.01) | -7.49 (-10.2, -4.78) | 0.41 |
| HDL-cholesterol, mg/dl |  |  |  |  |  |  |  |  |
| Model 1 | 1.29 ( 0.36, 2.23) | 1.44 ( 0.49, 2.39) | 1.78 ( 0.79, 2.77) | 1.55 ( 0.69, 2.42) | 0.59 | 1.51 ( 0.87, 2.15) | 1.53 ( 0.81, 2.24) | 0.97 |
| Model 2 | 1.73 ( 0.83, 2.63) | 1.54 ( 0.61, 2.47) | 1.57 ( 0.59, 2.55) | 1.30 ( 0.45, 2.15) | 0.54 | 1.57 ( 0.92, 2.22) | 1.49 ( 0.79, 2.18) | 0.87 |
| Model 3 | 1.69 ( 0.70, 2.68) | 1.57 ( 0.63, 2.50) | 1.62 ( 0.65, 2.59) | 1.26 ( 0.39, 2.13) | 0.59 | 1.54 ( 0.85, 2.23) | 1.53 ( 0.81, 2.25) | 0.98 |
| Triglycerides, mg/dl § |  |  |  |  |  |  |  |  |
| Model 1 | 0.91 ( 0.86, 0.96) | 0.94 ( 0.89, 0.98) | 0.91 ( 0.87, 0.95) | 0.94 ( 0.91, 0.98) | 0.43 | 0.93 ( 0.90, 0.96) | 0.92 ( 0.89, 0.96) | 0.91 |
| Model 2 | 0.89 ( 0.85, 0.94) | 0.93 ( 0.89, 0.97) | 0.92 ( 0.88, 0.96) | 0.96 ( 0.92, 1.00) | 0.07 | 0.93 ( 0.90, 0.96) | 0.91 ( 0.88, 0.95) | 0.45 |
| Model 3 | 0.89 ( 0.84, 0.94) | 0.93 ( 0.89, 0.97) | 0.92 ( 0.88, 0.96) | 0.96 ( 0.92, 1.00) | 0.08 | 0.93 ( 0.89, 0.96) | 0.92 ( 0.88, 0.96) | 0.76 |
| Fasting glucose, mg/dl |  |  |  |  |  |  |  |  |
| Model 1 | 3.18 ( 1.90, 4.45) | 6.28 ( 4.53, 8.04) | 3.63 ( 2.41, 4.85) | 4.23 ( 2.90, 5.55) | 0.60 | 5.17 ( 4.23, 6.11) | 3.20 ( 2.09, 4.31) | 0.004 |
| Model 2 | 2.46 ( 1.20, 3.73) | 6.21 ( 4.53, 7.89) | 4.07 ( 2.89, 5.25) | 4.69 ( 3.30, 6.08) | 0.06 | 4.70 ( 3.78, 5.63) | 3.86 ( 2.76, 4.96) | 0.19 |
| Model 3 | 2.64 ( 1.25, 4.04) | 6.13 ( 4.43, 7.82) | 3.84 ( 2.62, 5.05) | 4.83 ( 3.33, 6.34) | 0.15 | 4.80 ( 3.81, 5.79) | 3.73 ( 2.51, 4.96) | 0.18 |
| 2-h glucose, mg/dl |  |  |  |  |  |  |  |  |
| Model 1 | 3.82 ( 0.09, 7.55) | 10.26 ( 6.52, 14.01) | 6.40 ( 1.51, 11.29) | 5.96 ( 2.21, 9.71) | 0.57 | 6.27 ( 3.63, 8.90) | 7.02 ( 3.58, 10.46) | 0.73 |
| Model 2 | 2.70 (-1.08, 6.47) | 10.37 ( 6.61, 14.13) | 7.25 ( 2.70, 11.80) | 6.11 ( 2.40, 9.83) | 0.28 | 6.93 ( 4.24, 9.61) | 6.14 ( 3.01, 9.28) | 0.69 |
| Model 3 | 1.46 (-2.60, 5.53) | 10.24 ( 6.52, 13.96) | 7.44 ( 2.85, 12.04) | 7.34 ( 3.41, 11.26) | 0.09 | 7.09 ( 4.28, 9.89) | 5.93 ( 2.42, 9.44) | 0.62 |
| HbA1c, mg/dl |  |  |  |  |  |  |  |  |
| Model 1 | 0.20 ( 0.16, 0.24) | 0.26 ( 0.21, 0.31) | 0.12 ( 0.09, 0.16) | 0.18 ( 0.14, 0.21) | 0.05 | 0.18 ( 0.15, 0.20) | 0.20 ( 0.17, 0.24) | 0.28 |
| Model 2 | 0.18 ( 0.14, 0.22) | 0.26 ( 0.20, 0.31) | 0.14 ( 0.11, 0.17) | 0.19 ( 0.15, 0.22) | 0.38 | 0.18 ( 0.15, 0.20) | 0.21 ( 0.17, 0.25) | 0.23 |
| Model 3 | 0.17 ( 0.13, 0.21) | 0.25 ( 0.20, 0.31) | 0.14 ( 0.11, 0.17) | 0.20 ( 0.16, 0.24) | 0.99 | 0.19 ( 0.16, 0.21) | 0.20 ( 0.16, 0.24) | 0.70 |
| Fasting insulin, mU/L § |  |  |  |  |  |  |  |  |
| Model 1 | 1.17 ( 1.11, 1.24) | 1.30 ( 1.20, 1.42) | 1.16 ( 1.10, 1.23) | 1.18 ( 1.12, 1.24) | 0.63 | 1.22 ( 1.17, 1.27) | 1.19 ( 1.12, 1.25) | 0.41 |
| Model 2 | 1.16 ( 1.09, 1.23) | 1.29 ( 1.20, 1.39) | 1.18 ( 1.11, 1.24) | 1.19 ( 1.13, 1.26) | 0.75 | 1.21 ( 1.16, 1.26) | 1.20 ( 1.13, 1.26) | 0.77 |
| Model 3 | 1.17 ( 1.09, 1.24) | 1.29 ( 1.20, 1.39) | 1.16 ( 1.10, 1.23) | 1.19 ( 1.13, 1.26) | 0.98 | 1.21 ( 1.16, 1.27) | 1.19 ( 1.12, 1.26) | 0.62 |
| HOMA-IR § |  |  |  |  |  |  |  |  |
| Model 1 | 1.23 ( 1.15, 1.31) | 1.37 ( 1.25, 1.51) | 1.20 ( 1.13, 1.28) | 1.23 ( 1.15, 1.31) | 0.49 | 1.27 ( 1.22, 1.33) | 1.23 ( 1.16, 1.31) | 0.35 |
| Model 2 | 1.20 ( 1.12, 1.28) | 1.36 ( 1.25, 1.47) | 1.22 ( 1.15, 1.30) | 1.25 ( 1.18, 1.33) | 0.55 | 1.26 ( 1.21, 1.32) | 1.25 ( 1.18, 1.32) | 0.77 |
| Model 3 | 1.21 ( 1.13, 1.30) | 1.36 ( 1.25, 1.48) | 1.21 ( 1.14, 1.29) | 1.26 ( 1.18, 1.34) | 0.82 | 1.27 ( 1.21, 1.33) | 1.24 ( 1.17, 1.32) | 0.61 |
| *Meeting 2018 physical activity guidelines defined using measured activity scaled to 7 days of accelerometer wear as 150 minutes/week moderate intensity physical activity, 75 minutes/week vigorous intensity activity, or an equivalent combination of both.  Model 1 adjusted for age at baseline, sex, use of medications that affect the dependent variable at baseline and/or visit2, baseline levels of the dependent variable, and elapsed time between visits | | | | | | | | |
| Model 2 further adjusted for baseline household income, education, employment status, Hispanic/Latino background, field center, and nativity status, smoking, alcohol consumption, health insurance status, healthcare utilization, self-reported health, diet quality, change in health insurance, baseline BMI and waist-hip ratio (except in modeling change of BMI and waist circumference) | | | | | | | | |
| Model 3 adjusted for Model 2 covariates and sedentary time in models of MVPA or MVPA in models of sedentary time | | | | | | | | |
| §Geometric means (95% CI) presented for triglycerides, fasting insulin, HOMA-IR. | | | | | | | | |
