# Supplementary material for: Are sedentary behavior and physical activity independently associated with cardiometabolic benefits? The Hispanic Community Health Study/Study of Latinos
Source: BMC Public Health. 2020 Sep 14;20:1400. doi: 10.1186/s12889-020-09497-5 (PMC7490882; doi:10.1186/s12889-020-09497-5)
Supplement: Supplementary file 3 — Additional file 3: Table S3. LDL Cholesterol & Statin use. [file 12889_2020_9497_MOESM3_ESM.docx]

**Additional Table 3:** LDL Cholesterol & Statin use

| Statins V1 | Statins V2 | N | Mean change in LDL | P-value |
| --- | --- | --- | --- | --- |
| Yes | - | 1037 | -4.3 (27.6) | 0.003 |
| No | - | 6651 | -8.1 (38.6) |  |
| - | Yes | 1622 | -24.9 (38.5) | <0.0001 |
| - | No | 6066 | -2.9 (24.4) |  |
| Yes | Yes | 724 | -11.71 (37.0) | <0.0001 |
| Yes | No | 313 | 12.8 (36.9) |  |
| No | Yes | 898 | -35.6 (36.3) |  |
| No | No | 5753 | -3.8 (23.3) |  |
