# Supplementary material for: Are sedentary behavior and physical activity independently associated with cardiometabolic benefits? The Hispanic Community Health Study/Study of Latinos
Source: BMC Public Health. 2020 Sep 14;20:1400. doi: 10.1186/s12889-020-09497-5 (PMC7490882; doi:10.1186/s12889-020-09497-5)
Supplement: Supplementary file 4 — Additional file 4: Table S4. Multivariable-adjusted mean changes in cardiovascular disease risk factors (95% CI) over 6 years of follow-up, according to cross-classification of quartiles of sedentary time and meeting guidelines for moderate-to-vigorous physical activity. [file 12889_2020_9497_MOESM4_ESM.docx]

**Additional Table 4:** Multivariable-adjusted mean changes in cardiovascular disease risk factors (95% CI) over 6 years of follow-up, according to cross-classification of quartiles of sedentary time and meeting guidelines for moderate-to-vigorous physical activity*

|  | **Sedentary time** | | | |  |
| --- | --- | --- | --- | --- | --- |
| **Change in CVD risk factors** | **Quartile 1**  (0.8-10.8 h) | **Quartile 2**  (10.8-12.0 h) | **Quartile 3**  (12.0-13.0 h) | **Quartile 4**  (13.0-16.0 h) | ***P-int*** |
| BMI, kg/m2 |  |  |  |  | 0.56 |
| Not meet MVPA guideline | 0.43 ( 0.10, 0.76) | 0.67 ( 0.43, 0.92) | 0.56 ( 0.34, 0.77) | 0.73 ( 0.51, 0.94) |  |
| Meet MVPA guideline | 0.76 ( 0.53, 0.98) | 0.91 ( 0.63, 1.20) | 0.52 ( 0.17, 0.87) | 0.73 ( 0.31, 1.15) |  |
| Waist circumference, cm |  |  |  |  | 0.58 |
| Not meet MVPA guideline | 2.00 ( 1.10, 2.91) | 2.12 ( 1.37, 2.87) | 2.01 ( 1.31, 2.71) | 2.64 ( 2.06, 3.22) |  |
| Meet MVPA guideline | 2.54 ( 1.93, 3.15) | 2.48 ( 1.66, 3.30) | 1.54 ( 0.59, 2.49) | 2.37 ( 1.10, 3.63) |  |
| Systolic BP, mmHg |  |  |  |  | 0.47 |
| Not meet MVPA guideline | 1.49 ( 0.00, 2.97) | 1.87 ( 0.73, 3.00) | 0.69 (-0.32, 1.71) | 0.50 (-0.43, 1.42) |  |
| Meet MVPA guideline | 0.66 (-0.31, 1.62) | 0.96 (-0.20, 2.12) | 1.36 (-0.05, 2.78) | 0.78 (-0.89, 2.46) |  |
| Diastolic BP, mmHg |  |  |  |  | 0.32 |
| Not meet MVPA guideline | 0.60 (-0.58, 1.78) | 0.56 (-0.16, 1.27) | -0.20 (-0.96, 0.57) | -0.50 (-1.25, 0.24) |  |
| Meet MVPA guideline | -0.19 (-0.91, 0.54) | 0.49 (-0.44, 1.42) | 0.44 (-0.54, 1.41) | 0.22 (-1.17, 1.61) |  |
| LDL-cholesterol, mg/dl |  |  |  |  | 0.66 |
| Not meet MVPA guideline | -5.96 (-8.48, -3.44) | -3.13 (-5.44, -0.82) | -3.43 (-5.87, -1.00) | -4.76 (-7.07, -2.46) |  |
| Meet MVPA guideline | -5.38 (-7.38, -3.39) | -4.58 (-7.79, -1.37) | -5.76 (-8.97, -2.55) | -4.88 (-8.40, -1.36) |  |
| HDL-cholesterol, mg/dl |  |  |  |  | 0.49 |
| Not meet MVPA guideline | 1.64 ( 0.26, 3.03) | 1.84 ( 1.12, 2.55) | 2.04 ( 1.28, 2.79) | 1.46 ( 0.75, 2.17) |  |
| Meet MVPA guideline | 1.61 ( 0.87, 2.36) | 0.83 (-0.06, 1.73) | 2.00 ( 0.64, 3.36) | 0.12 (-1.39, 1.62) |  |
| Triglycerides, mg/dl § |  |  |  |  | 0.76 |
| Not meet MVPA guideline | 0.93 ( 0.87, 0.98) | 0.92 ( 0.89, 0.96) | 0.94 ( 0.91, 0.98) | 0.94 ( 0.90, 0.98) |  |
| Meet MVPA guideline | 0.96 ( 0.92, 1.00) | 0.93 ( 0.88, 0.99) | 0.94 ( 0.89, 0.99) | 0.99 ( 0.92, 1.06) |  |
| Fasting glucose, mg/dl |  |  |  |  | 0.06 |
| Not meet MVPA guideline | 4.76 ( 3.16, 6.37) | 4.86 ( 3.54, 6.19) | 3.81 ( 2.45, 5.17) | 4.92 ( 3.74, 6.09) |  |
| Meet MVPA guideline | 3.10 ( 1.95, 4.26) | 3.36 ( 2.13, 4.59) | 5.10 ( 3.67, 6.52) | 3.50 ( 1.28, 5.73) |  |
| 2-hour glucose, mg/dl |  |  |  |  | 0.80 |
| Not meet MVPA guideline | 8.19 ( 4.10, 12.28) | 8.00 ( 4.99, 11.01) | 6.67 ( 3.36, 9.99) | 9.98 ( 7.12, 12.84) |  |
| Meet MVPA guideline | 6.57 ( 3.82, 9.32) | 8.32 ( 4.23, 12.42) | 8.83 ( 3.84, 13.83) | 9.58 ( 5.35, 13.82) |  |
| HbA1c, mg/dl |  |  |  |  | 0.63 |
| Not meet MVPA guideline | 0.20 ( 0.14, 0.25) | 0.18 ( 0.14, 0.21) | 0.15 ( 0.11, 0.19) | 0.15 ( 0.12, 0.19) |  |
| Meet MVPA guideline | 0.18 ( 0.15, 0.21) | 0.19 ( 0.15, 0.23) | 0.18 ( 0.13, 0.22) | 0.19 ( 0.13, 0.25) |  |
| Fasting insulin, mU/L § |  |  |  |  | 0.90 |
| Not meet MVPA guideline | 1.20 ( 1.13, 1.27) | 1.23 ( 1.17, 1.28) | 1.22 ( 1.16, 1.28) | 1.23 ( 1.19, 1.28) |  |
| Meet MVPA guideline | 1.17 ( 1.11, 1.23) | 1.22 ( 1.14, 1.31) | 1.17 ( 1.08, 1.25) | 1.20 ( 1.11, 1.31) |  |
| HOMA-IR § |  |  |  |  | 0.97 |
| Not meet MVPA guideline | 1.25 ( 1.18, 1.33) | 1.28 ( 1.21, 1.35) | 1.27 ( 1.20, 1.34) | 1.29 ( 1.23, 1.34) |  |
| Meet MVPA guideline | 1.22 ( 1.16, 1.29) | 1.27 ( 1.18, 1.37) | 1.22 ( 1.13, 1.32) | 1.27 ( 1.16, 1.39) |  |
| *All models adjusted for sex, use of medications that affect the dependent variable at baseline and/or visit2, baseline levels of the dependent variable, and elapsed time between visits, household income, education, employment status, Hispanic/Latino background, field center, and nativity status, smoking, alcohol consumption, health insurance status, healthcare utilization, self-reported health, diet quality (all assessed at baseline), change in health insurance coverage, BMI and waist-to-hip ratio (except for change in BMI and waist circumference as outcomes). Meeting 2018 physical activity guidelines defined using measured activity scaled to 7 days of accelerometer wear as 150 minutes/week moderate intensity physical activity, 75 minutes/week vigorous intensity activity, or an equivalent combination of both. §Geometric means (95% CI) presented for triglycerides, fasting insulin, HOMA-IR. | | | | | |
